# Efficacy and safety of different drugs for the treatment of leishmaniasis: a systematic review and network meta-analysis

**DOI:** 10.3389/fcimb.2025.1717863

**Published:** 2025-12-15

**Authors:** Yuxin Fan, Yanhong Gu, Xiaodong Tong, Youli He, Qinya Li, Kaiyang Chen, Zhiwei Cui, Yang Yang, Yin Xiang, Qi Liu

**Affiliations:** 1 Department of Medical Laboratory, The People’s Hospital of Leshan, Leshan, China; 2 Department of Medical Laboratory, Emeishan City People’s Hospital, Leshan, China; 3 College of Life Science and Technology, Kunming University of Science and Technology, Kunming, China

**Keywords:** leishmaniasis, efficacy, safety, network meta-analysis, amphotericin B

## Abstract

**Objectives:**

Leishmaniasis is a vector-borne neglected tropical infectious disease hat can be fatal if visceral leishmaniasis is left untreated. At present, many drugs have been used to treat leishmaniasis. This study aims to compare the efficacy and safety of different drugs to treat leishmaniasis through a network meta-analysis.

**Methods:**

All studies were retrieved from PubMed, Embase, and Cochrane databases, and the search time was from the date of database establishment to February 21, 2024. We assessed rates of clinical cure, mortality, and adverse effects (diarrhea, vomiting, injection site pain, and liver-enzyme abnormalities). We performed subgroup analyses of rates of clinical cure according to geographic region. All statistical analyses were performed using R and STATA 14.0 software for network meta-analysis. PROSPERO registration number: CRD42023478585.

**Results:**

12 articles with 2483 patients were included in this study, and the therapeutic effects of 5 drugs were evaluated. The results of the network Meta-analysis showed that the clinical cure rate of miltefosine, pentavalent antimony, and paromomycin was inferior to amphotericin B (RR 0.31; 95%CI 0.07-11.4, RR 0.23; 95%CI 0.04-1.39, RR 0.12; 95% CI 0.01-1.55), and amphotericin B may have the highest clinical cure rate. The mortality of pentavalent antimony was higher than that of amphotericin B and miltefosine (RR 4.81; 95%CI 0.42-41.45, RR 3.75; 95% CI 0.57-24.74), pentavalent antimony may be the drug with the highest mortality during treatment. About adverse effects, vomiting, and diarrhea were most common with miltefosine, and pain at the injection site and abnormalities in aspartate aminotransferase and alanine aminotransferase were most common with paromomycin. Subgroup analysis showed that clinical cure rates using Pentavalent antimony treatment were better in Brazil than in Ethiopia and India.

**Conclusions:**

This systematic review and network meta-analysis provide a new comparative framework for the clinical management of leishmaniasis. The results of network meta-analysis suggest that amphotericin B may be the first choice for the treatment of leishmaniasis, pentavalent antimony may be the drug with the highest mortality during treatment, miltefosine causes the highest incidence of gastrointestinal reactions, and paromomycin causes the highest incidence of injection site pain, aspartate aminotransferase, and alanine aminotransferase abnormalities. More high-quality studies are still needed to further determine the optimal drug for the clinical treatment of leishmaniasis in the future.

**Systematic Review Registration:**

https://www.crd.york.ac.uk/prospero/, identifier CRD42023478585.

## Introduction

Neglected tropical diseases (NTDS) are a group of diseases that are strongly associated with poverty and occur mainly in tropical and subtropical countries. Leishmaniasis is a tropical infectious disease caused by *Leishmania* spp, which is listed as a NTDS by the World Health Organization (WHO), 2010 (Uliana et al., 2018). There are about 0.7 to 1.2 million new cases of leishmaniasis every year, putting nearly 350 million people at risk of infection globally (Burza et al., 2019; Kumari et al., 2021). Visceral leishmaniasis (VL) is most severe in East Africa, where it accounts for 45% of cases globally (Alvar et al., 2021). Cutaneous leishmaniasis (CL) is susceptible in children, and the median age of adult patients is 37 years, with a male proportion of 68% (Van der Auwera et al., 2022; Doni and An, 2024).

Leishmaniasis is mainly divided into cutaneous leishmaniasis and visceral leishmaniasis, of which VL is the most severe presentation and ranks second only to malaria in the global burden of parasitic diseases (Uliana et al., 2018). The clinical manifestations of VL are characterized by persistent irregular fever and splenomegaly, pancytopenia, hepatomegaly, hypergammaglobulinemia, and weight loss (Alvar et al., 2021), which can lead to death if left untreated (Diro et al., 2014). To date, there is no vaccine available to prevent leishmaniasis in humans (Burza et al., 2018). pentavalent antimony, amphotericin B, miltefosine, paromomycin, paromomycin ketoconazole, and so on are the main drugs for leishmaniasis. Among them, pentavalent antimony, after being converted to trivalent antimony *in vivo*, plays a killing role by interfering with energy metabolism and inhibiting DNA topoisomerase I activity of Leishmania (Gutierrez Guarnizo et al., 2021). Amphotericin B, by binding to ergosterol on protozoan cell membranes, forms transmembrane ion channels that disrupt cell membrane permeability and ion balance, leading to cell death due to leakage of cell contents (Queffeulou et al., 2024; Tulloch et al., 2024). Miltefosine interferes with cell membrane phospholipid metabolism by inhibiting phosphatidylcholine synthetase (Queffeulou et al., 2024). At the same time, apoptosis-like death is induced by transporter entry into the parasite (Ware et al., 2021). As an aminoglycoside antibiotic, paromomycin interferes with protein synthesis by binding to the 30S subunit of the protozoan ribosome (de Sa et al., 2023; Musa et al., 2023). Ketoconazole inhibits cytochrome P450 such as CYP51, blocks ergosterol synthesis, and affects membrane structural integrity (Jin et al., 2024). But these drugs have different degrees of side effects and drug resistance, which hinder the success of the treatment of leishmaniasis (Garza-Tovar et al., 2020). Although many new therapeutic approaches are emerging, such as host-directed therapy, drug reuse, and nanotechnology, most of them are still in the research stage, and the requirements of sensitivity, preparation, and operation of nanotechnology limit their use (Garza-Tovar et al., 2020; Kumari et al., 2021). The elimination of leishmaniasis still faces many challenges. Due to global climate change, urbanization, deforestation, and co-infection, leishmaniasis has re-emerged in local areas. At present, the treatment of leishmaniasis still relies on chemotherapy drugs, so the efficacy and safety of drugs are particularly important for the selection of clinical drugs.

In this study, we conducted a network meta-analysis (NMA) to compare the efficacy and safety of different drugs (pentavalent antimony, amphotericin B, miltefosine, paromomycin, ketoconazole) in the treatment of leishmaniasis, to determine which drug is the best choice for the treatment of leishmaniasis, and to provide more evidence for the clinical use of leishmaniasis.

## Methods

### Search strategy

We conducted a systematic search in PubMed, EMBASE and Cochrane databases using Boolean operators to identify studies that might meet the criteria (from From 1992 to February 2025). The aim was to retrieve two categories: leishmaniasis and therapeutic drugs. In addition, the references of all included studies and those that had undergone a meta-analysis on the same topic were manually searched for additional eligible studies(See **Supplementary Tables S1**–**S3**).

### Selection criteria

To be included in this study, articles had to meet the following criteria: 1. Randomized controlled trial (RCT) or observational study published in English. 2. The subjects were parasitologically confirmed leishmaniasis patients, mainly including CL and VL; 3. The effects of five drugs (pentavalent antimony, amphotericin B, miltefosine, paromomycin, and ketoconazole) on leishmaniasis were compared with or with each other. 4. The primary outcomes included the rate of clinical cure at the test-of-cure visit, mortality, and the rate of adverse effects.

### Data extraction

For each included study, data extraction was performed by two independent reviewers using a standardized data extraction form, and relevant study characteristics extracted included: 1. The first author; 2. Year of publication; 3. Research types; 4. Region; 5. Types of leishmaniasis infection; 6. Basic information of patients; 7. Medication information (medication method, dosage, and duration); 8. Efficacy (clinical cure rate); 9. Mortality; 10. Adverse reactions (Alanine aminotransferase/Aspartate aminotransferase abnormality, Diarrhea, Injection site pain, Vomiting).

### Outcome measures

We considered three outcomes: clinical cure rate, mortality, and adverse events (AE) in NMA. Clinical success is to assess the status of such a study population on the test of cure (TOC), defined as cure or improvement. The cure was defined as clinical improvement at the end of treatment, clearance of parasites from spleen, bone marrow, or lymph node tissue aspirates, and no recurrence during follow-up. Finally, safety was assessed in terms of the incidence of adverse events (diarrhea, vomiting, pain at the injection site, and liver-enzyme abnormalities) and mortality.

### Quality evaluation

The quality of the included studies was assessed by two independent reviewers according to the tools provided in the Cochrane Handbook, and when necessary, disagreements were resolved by two independent reviewers through discussion with a third reviewer. Finally, Review Manager 5.4 software was used to generate the risk of bias map.

### Statistical analysis

We compared the efficacy and safety of various drugs for leishmaniasis by evaluating the three outcome measures as well as the clinical cure rate of treatment with the same drugs in different countries using the NMA. Stata 14.0 software and R software were used to analyze the data. This study analyzed all available data using a random effects model to obtain the relative risk (RR) and 95% confidence interval (95% CI) comparing the efficacy and safety of various drugs in the designated population. The funnel plot was used to evaluate the publication bias of the included studies. This study was registered with PROSPERO under the number CRD42023478585.

## Results

### Study selection

**Figure 1:**
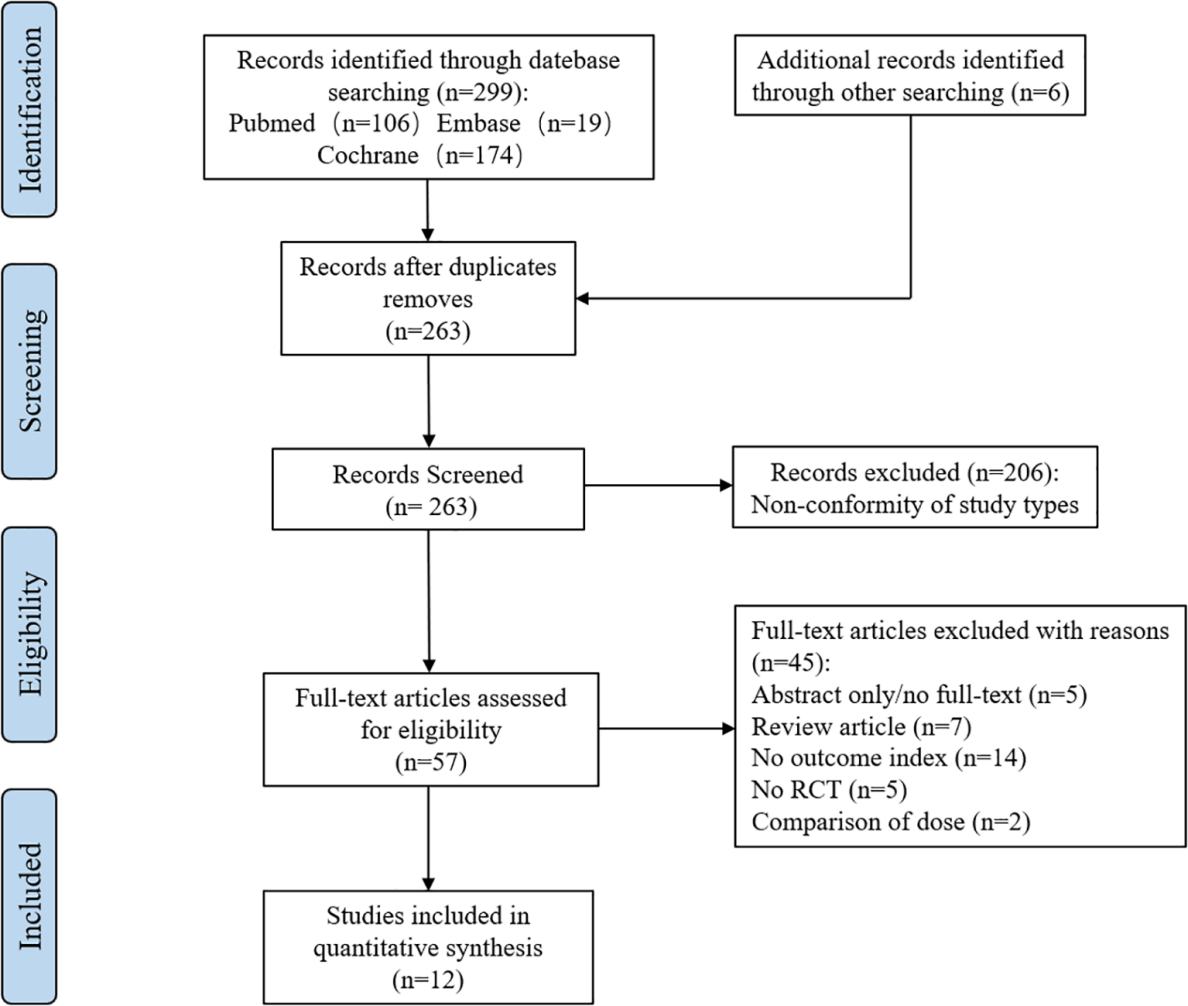
Literature screening flowchart.

We searched PubMed, Embase, and Cochrane databases for a total of 299 potentially relevant articles. After excluding 42 duplicate articles, the full-text contents of 57 articles were obtained by reading the titles and abstracts. These articles were evaluated in full text, and finally, we included 12 eligible studies that met the criteria (see **Figure 1**).

### Research characteristics

The main characteristics of the included studies are shown in **Table 1**, and the 12 articles included a total of 2483 patients. All study participants were parasitologically confirmed leishmaniasis patients and five drugs were evaluated as monotherapy for the treatment of leishmaniasis patients.

**Table 1:** Characteristics of the included studies (RCT, randomized controlled trial; CR, cure rate; AR, adverse reaction; MR, mortality rate).

Study	Country	Study design	Interventions	Sample size	Dosage	Outcomes
(Thakur et al., 1993)	India	RCT	Amphotericin B	75	1 mg/kg for 30 days	CR, AR
			Pentavalent antimony	75	20 mg/kg for 30 days	
(Mishra et al., 1994)	India	RCT	Pentavalent antimony	40	20mg/kg for 40 days	CR
			Amphotericin B	40	0.5mg/kg for 28 days	
(Wali et al., 1997)	India	RCT	Ketoconazole	26	600 mg/d for 28 days	CR
			Pentavalent antimony	78	20 mg/kg for 28 days	
(Thakur et al., 1997)	India	Unclear	Amphotericin B	11	1mg/kg/for 20 days	CR, AR
			Pentavalent antimony	11	20mg/kg for 20days	
(Sundar et al., 2002)	India	RCT, open-label	Miltefosine	299	2.5 mg/kg for 28 days	CR, AR
			Amphotericin B	99	1 mg/kg for 15 days	
(Thakur and Narayan, 2004)	India	Unclear	Amphotericin B	60	1mg/kg for 20 days	CR, AR, MR
			Pentavalent antimony	60	20mg/kg for 28days	
(Singh et al., 2006)	India	RCT, open-label	Miltefosine	44	2.5 mg/kg for 15 days	CR, AR
			Amphotericin B	38	1 mg/Kg for 15 days	
(Ritmeijer et al., 2006)	Ethiopia	RCT, open-label	Miltefosine	131	100 mg/d for 28 days	CR, AR, MR
			Pentavalent antimony	137	20 mg/kg for 28 days	
(Sundar et al., 2007)	India	RCT, open-label	Paromomycin	501	11 mg/kg for 21 days	CR, AR, MR
			Amphotericin B	165	1 mg/kg for 30 days	
(Musa et al., 2012)	Multinational	RCT	Pentavalent antimony	200	20mg/kg for 30 days	CR, AR, MR
			Paromomycin	198	20mg/kg for 21days	
(Borges et al., 2017)	Brazil	RCT, open-label	Pentavalent antimony	51	20mg/kg for 20 days	CR, AR
			Amphotericin B	51	1mg/kg for 14 days	
(Pandey et al., 2021)	India	RCT, open-label	Amphotericin B	47	5mg/kg for 3 weeks	CR, AR
			Miltefosine	46	2.5mg/kg for 12 weeks	

### Bias risk assessment

Among the 12 included studies, 9 studies were clearly defined as RCTS, and the other 3 studies did not clearly state that there was a certain high risk of bias. In general, the risk of bias was low to moderate in all included studies (see **Supplementary Figures S1**, **S2**).

### Clinical cure rate

**Figure 2:**
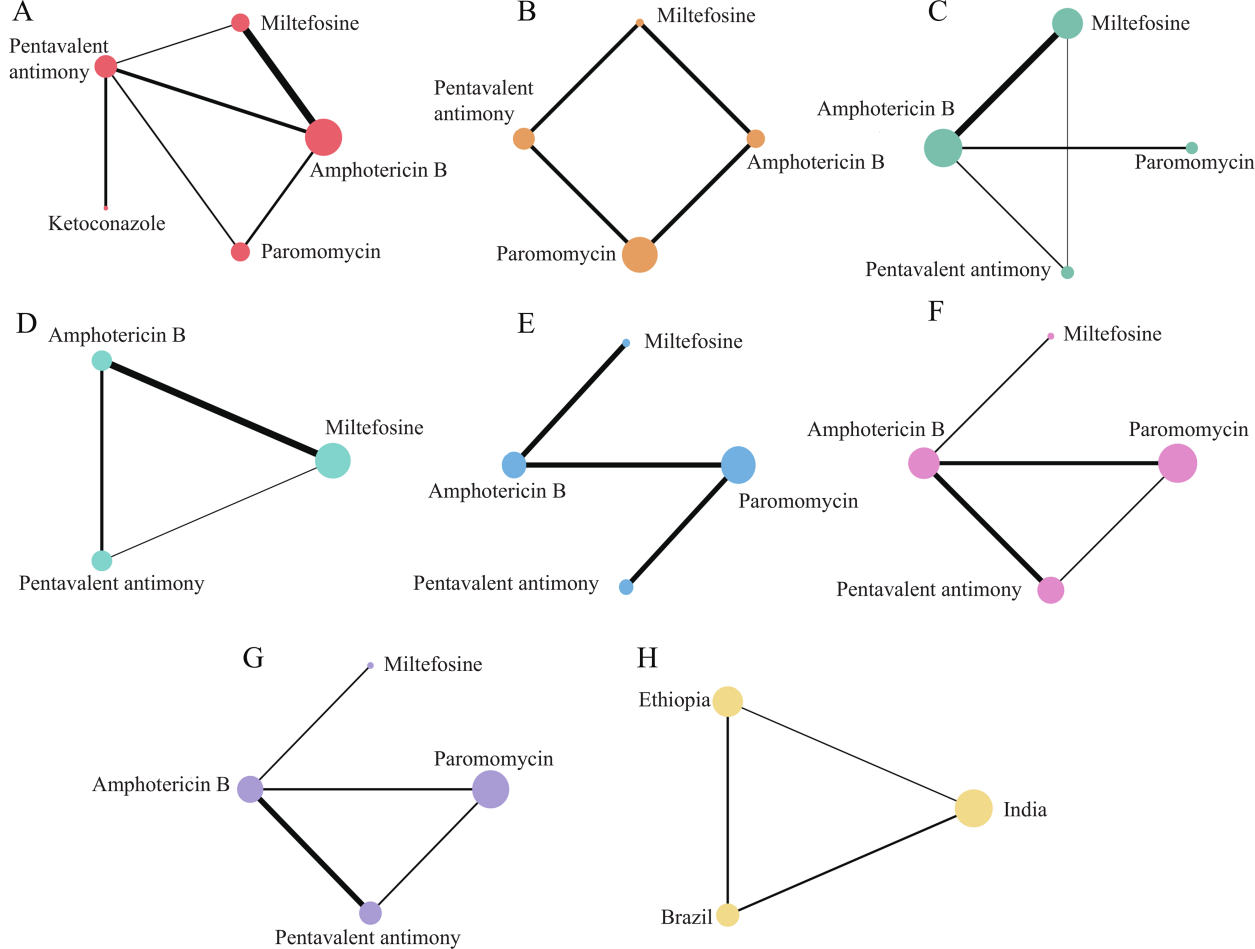
Network evidence plot, where the size of nodes corresponds to the cumulative sample size for individual antibiotics, and the thickness of the lines is proportional to the number of studies for each treatment comparison. **(A)** Clinical cure rate. **(B)** Mortality. **(C)** Incidence of adverse reactions (Vomiting). **(D)** Incidence of adverse reactions (Diarrhea). **(E)** Incidence of adverse reactions (Injection site pain). **(F)** Incidence of adverse reactions (Alanine aminotransferase abnormality). **(G)** Incidence of adverse effects (Aspartate aminotransferase abnormality) **(H)** Cure rates of pentavalent antimony in three different countries.

**Figure 3:**
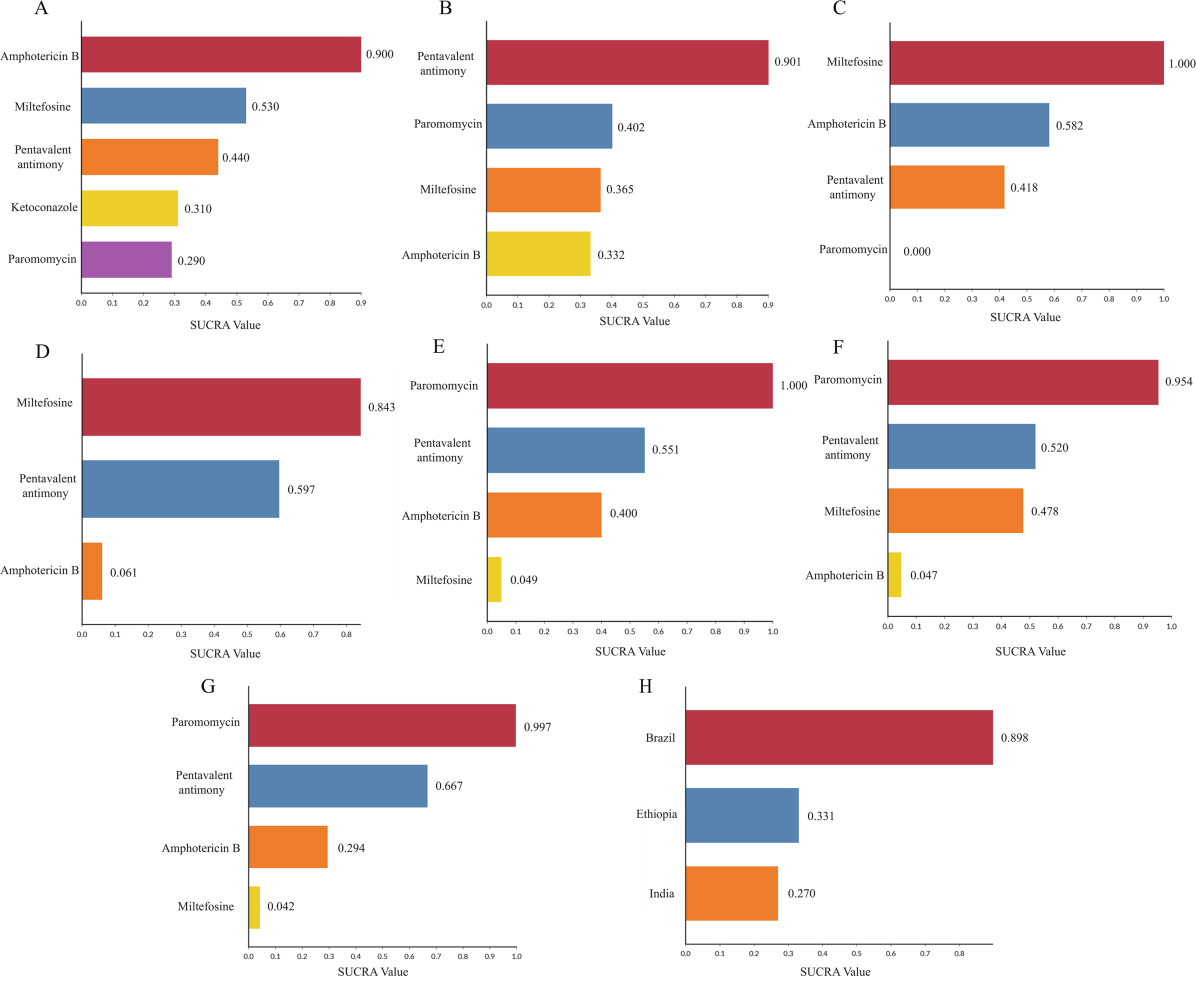
Efficacy and safety of drugs for the treatment of leishmaniasis: **(A)** Clinical cure rate. **(B)** Mortality. **(C)** Incidence of adverse reactions (vomiting). **(D)** Incidence of adverse reactions (diarrhea). **(E)** Incidence of adverse reactions (pain at injection site). **(F)** Incidence of adverse effects (abnormal alanine aminotransferase). **(G)** Incidence of adverse effects (abnormal aspartate aminotransferase). **(H)** Cure rates of pentavalent antimony in three different countries.SUCRA value: Surface Under the Cumulative Ranking Curve value.

In the 12 included articles, the clinical cure rate of 2483 patients was evaluated, of which 2229 patients were cured. To further determine the direct and indirect comparative efficacy between the five interventions, we conducted a network meta-analysis, and the network evidence graph is shown in **Figure 2A**. The evidence map directly or indirectly compares the treatment effects of the five interventions. The results showed that the clinical cure rate of miltefosine was inferior to amphotericin B (RR 0.31; 95% CI 0.07-11.48). The clinical cure rate of pentavalent antimony was inferior to amphotericin B (RR 0.23; 95% CI 0.04-1.39). The clinical cure rate of paromomycin was inferior to that of amphotericin B (RR 0.12; 95% CI 0.01-1.55) (See **Table 2** and **Supplementary Figure S3**). We ranked the drugs according to the probability of clinical cure success. SUCRA results showed that amphotericin B (92%) ranked first, followed by miltefosine (53.2%), pentavalent antimony (44.3%), ketoconazole (31.5%), and paromomycin (29%). Rank probability plots and rank cumulative probability plots for all therapeutic drugs are shown in **Figure 3A** and **Supplementary Figures S11**.

**Table 2:** Pairwise comparison of the efficacy and safety of drugs for the treatment of leishmaniasis.

Comparisons	Network meta-analysis	
	RR (95% CI)	Certainty of evidence
A. Efficacy of drugs used to treat leishmaniasis		
Miltefosine vs Amphotericin B	0.31 (0.07,1.48)	⊕○○○
Pentavalent antimony vs Amphotericin B	0.23 (0.04,1.39)	⊕○○○
Ketoconazole vs Amphotericin B	0.11 (0.00,6.18)	⊕⊕○○
Paromomycin vs Amphotericin B	0.12 (0.01,1.55)	⊕○○○
Pentavalent antimony vs Miltefosine	0.72 (0.10,5.38)	⊕○○○
Ketoconazole vs Miltefosine	0.34 (0.01,21.52)	⊕⊕○○
Paromomycin vs Miltefosine	0.40 (0.02,6.59)	⊕○○○
Ketoconazole vs Pentavalent antimony	0.48 (0.01,17.76)	⊕○○○
Paromomycin vs Pentavalent antimony	0.55 (0.05,6.60)	⊕○○○
Paromomycin vs Ketoconazole	1.15 (0.01,91.84)	⊕⊕○○
B. Mortality of drugs used to treat leishmaniasis		
Miltefosine vs Amphotericin B	1.12 (0.14,8.75)	⊕⊕○○
Pentavalent antimony vs Amphotericin B	4.18 (0.42,41.45)	⊕⊕⊕○
Paromomycin vs Amphotericin B	1.20 (0.15,9.28)	⊕⊕⊕○
Pentavalent antimony vs Miltefosine	3.75 (0.57,24.74)	⊕⊕⊕○
Paromomycin vs Miltefosine	1.07 (0.11,10.49)	⊕⊕○○
Paromomycin vs Pentavalent antimony	0.29 (0.04,1.97)	⊕⊕○○
C. Incidence of adverse reactions to drugs used to treat leishmaniasis -Vomiting		
Miltefosine vs Paromomycin	41.45 (11.16,153.89)	⊕○○○
Amphotericin B vs Paromomycin	17.66 (5.08,61.41)	⊕○○○
Pentavalent antimony vs Paromomycin	15.16 (4.00,57.39)	⊕○○○
Amphotericin B vs Miltefosine	0.43 (0.28,0.64)	⊕○○○
Pentavalent antimony vs Miltefosine	0.37 (0.27,0.50)	⊕○○○
Pentavalent antimony vs Amphotericin B	0.86 (0.54,1.37)	⊕○○○
D. Incidence of adverse reactions to drugs used to treat leishmaniasis -Diarrhea		
Amphotericin B vs Miltefosine	0.18 (0.04,0.94)	⊕○○○
Pentavalent antimony vs Miltefosine	0.61 (0.09,4.05)	⊕○○○
Pentavalent antimony vs Amphotericin B	3.31 (0.55,19.88)	⊕○○○
E. Incidence of adverse reactions to drugs used to treat leishmaniasis -Injection site pain		
Miltefosine vs Paromomycin	0.00 (0.00,0.01)	⊕⊕○○
Amphotericin B vs Paromomycin	0.00 (0.00,0.04)	⊕⊕○○
Pentavalent antimony vs Paromomycin	0.01 (0.00,0.14)	⊕⊕⊕○
Amphotericin B vs Miltefosine	9.76 (0.51,186.11)	⊕○○○
Pentavalent antimony vs Miltefosine	33.54 (0.24,4629.58)	⊕○○○
Pentavalent antimony vs Amphotericin B	3.44 (0.07,178.27)	⊕○○○
F. Incidence of adverse reactions of drugs used to treat leishmaniasis -abnormal Alanine aminotransferase		
Miltefosine vs Paromomycin	0.48 (0.14,1.67)	⊕○○○
Amphotericin B vs Paromomycin	0.27 (0.11,0.66)	⊕○○○
Pentavalent antimony vs Paromomycin	0.55 (0.34,0.89)	⊕○○○
Amphotericin B vs Miltefosine	0.57 (0.24,1.38)	⊕○○○
Pentavalent antimony vs Miltefosine	1.15 (0.35,3.76)	⊕○○○
Pentavalent antimony vs Amphotericin B	2.01 (0.92,4.43)	⊕○○○
H. Incidence of adverse reactions to drugs used to treat leishmaniasis -Aspartate aminotransferase abnormalities		
Miltefosine vs Paromomycin	0.13 (0.04,0.41)	⊕○○○
Amphotericin B vs Paromomycin	0.22 (0.10,0.46)	⊕○○○
Pentavalent antimony vs Paromomycin	0.57 (0.36,0.91)	⊕○○○
Amphotericin B vs Miltefosine	1.68 (0.70,4.05)	⊕○○○
Pentavalent antimony vs Miltefosine	4.44 (1.43,13.76)	⊕○○○
Pentavalent antimony vs Amphotericin B	2.64 (1.30,5.39)	⊕○○○
I. Efficacy of pentavalent antimony in the treatment of leishmaniasis in three different countries		
Ethiopia vs India	1.14 (0.15,8.39)	⊕○○○
Brazil vs India	5.33 (0.45,63.14)	⊕○○○
Brazil vs Ethiopia	4.68 (0.40,54.90)	⊕○○○

RR, Relative risk; 95% CI, 95% confidence interval. If the range of 95% CI includes the threshold value (the threshold value of RR is 1), it indicates the difference of comparison is not significant. The certainty of the evidence (according to GRADE) is incorporated in this table and categorized as “high” (⊕⊕⊕⊕), “moderate” (⊕⊕⊕○), “low” (⊕⊕○○), or “very low” (⊕○○○).

In addition, among the included 12 articles, a total of 7 articles evaluated the clinical cure rate of 452 patients with leishmaniasis treated with pentavalent antimony, of which 352 patients were successfully cured. Seven studies included 452 patients from India, Brazil, and Ethiopia. To further compare the efficacy of pentavalent antimony in the treatment of leishmaniasis in three different countries, we conducted a subgroup analysis (**Figure 2H**). The NMA results showed that the efficacy of Pentavalent antimony treatment in Brazil was better than that in India (RR 5.33; 95% CI 0.45-63.14). The efficacy of pentavalent antimony treatment in Brazil was better than that in Ethiopia (RR 4.68; 95% CI 0.40-54.90) (See **Table 2** and **Supplementary Figure S10**). We ranked the three countries according to the probability of efficacy of Pentavalent antimony treatment for leishmaniasis. The SUCRA results showed that Brazil (89.8%) had the highest probability of first efficacy of pentavalent antimony treatment, followed by Ethiopia (33.1%) and India (27%). Rank probability plots for all regions as well as rank cumulative probability plots are shown in **Figure 3H** and **Supplementary Figures S18**.

### Mortality rate

Of the 12 included articles, a total of 4 articles evaluated the mortality of 1645 patients, of which 17 patients died. The NMA compares the direct and indirect comparative efficacy of four interventions (pentavalent antimony, amphotericin B, miltefosine, and paromomycin), and the network evidence graph is shown in **Figure 2B**. NMA results showed that the mortality rate of pentavalent antimony was higher than that of amphotericin B (RR 4.81; 95% CI 0.42-41.45). The mortality rate of pentavalent antimony was higher than that of miltefosine (RR 3.75; 95% CI 0.57-24.74). The mortality rate of paromomycin was lower than that of pentavalent antimony (RR 0.29; 95% CI 0.04-1.97) (see **Table 2** and **Supplementary Figure S4**). The SUCRA results showed that pentavalent antimony (90.1%) ranked first, followed by paromycin (40.2%), amphotericin (36.5%), and miltefosine (33.2%). Rank probability plots and rank cumulative probability plots for all drugs are shown in **Figure 3B** and **Supplementary Figures S12**.

### Incidence of adverse effects-gastrointestinal reactions

Vomiting and Diarrhea are common gastrointestinal adverse effects in the treatment of leishmaniasis. Among the 12 included articles, 6 articles evaluated the incidence of adverse reactions of vomiting in 1928 patients, of which 511 patients had vomiting symptoms, involving 4 interventions (pentavalent antimony, miltefosine, amphotericin B, paromomycin) (**Figure 2C**). The NMA results showed that the incidence of Vomiting was higher in miltefosine than in paromomycin (RR 41.45; 95% CI 11.16-153.89). The Vomiting rate of amphotericin B was higher than that of paromomycin (RR17.66; 95% CI 5.08-61.41). The incidence of Vomiting in pentavalent antimony was higher than that in paromomycin (RR 15.16; 95% CI 4.00-57.39), and amphotericin B had a lower incidence of Vomiting than miltefosine (RR 0.43; 95% CI 0.28-0.64). The incidence of adverse reactions in pentavalent antimony was inferior to that in miltefosine (RR 0.37; 95% CI 0.27-0.50) (see **Table 2** and **Supplementary Figure S5**). We ranked the incidence rate of antibiotics according to the probability of Vomiting, and the results showed that miltefosine (100%) ranked first place, followed by amphotericin B (58.2%), pentavalent antimony (41.8%), and paromomycin (0%). Rank probability plots and rank cumulative probability plots for all drugs are shown in **Figure 3C** and **Supplementary Figure S13**.

Among the 12 included studies, 5 studies evaluated the incidence of adverse reactions of diarrhea in 1253 patients, of which 396 patients had symptoms of diarrhea, involving 3 interventions (pentavalent antimony, amphotericin B, miltefosine). The NMA further determined the direct and indirect comparative efficacy of these 3 interventions, and the network evidence graph is shown in **Figure 2D**. The incidence of Diarrhea with amphotericin B was inferior to miltefosine (RR 0.18; 95% CI, 0.04 to 0.94) (**Table 2**; **Supplementary Figure S6**). The interventions were ranked according to their probability of incidence of Diarrhea, with miltefosine (84.7%) treatment ranking first, followed by pentavalent antimony (59.7%) and amphotericin B (6.1%). Rank probability plots and rank cumulative probability plots for all drugs are shown in **Figure 3D** and **Supplementary Figure S14**.

### Incidence of adverse effects on liver enzymes

Among the 12 included articles, 5 articles evaluated the incidence of adverse reactions of abnormal aspartate aminotransferase (AST) and alanine aminotransferase (ALT) in 1561 patients, of which 187 patients had abnormal aspartate aminotransferase and 160 patients had abnormal alanine aminotransferase. Four interventions (pentavalent antimony, amphotericin B, miltefosine, and paromomycin) were involved. The NMA further determined the direct and indirect comparative effectiveness of the four interventions; the network evidence plots are shown in **Figures 2F, G**. The NMA results showed that the incidence of AST abnormalities was lower with miltefosine than with paromomycin (RR 0.13; 95% CI 0.04-0.41), and amphotericin B had a lower incidence of abnormal AST than paromomycin (RR 0.22; 95% CI 0.10-0.46). The incidence of abnormal AST in pentavalent antimony was inferior to that in paromomycin (RR 0.57; 95% CI 0.36-0.91). The incidence of AST abnormality in pentavalent antimony was higher than that in miltefosine (RR 4.44; 95% CI 1.43-13.76). The incidence of AST abnormality in pentavalent antimony was higher than that in amphotericin B (RR 2.64; 95% CI 1.30-5.39) (**Table 2** and **Supplementary Figure S9**). SUCRA results showed that paromomycin (99.7%) was the most common treatment, followed by pentavalent antimony (66.7%), amphotericin B (29.4%), and miltefosine (4.2%). Rank probability plots and rank cumulative probability plots for all drugs are shown in **Figure 3G**; **Supplementary Figures S17**.

In terms of adverse effects of abnormal ALT, NMA results showed that the incidence of adverse effects of amphotericin B was lower than that of paromomycin (RR 0.27; 95% CI 0.11-0.66). The incidence of adverse reactions of pentavalent antimony was lower than that of paromomycin (RR 0.55; 95% CI 0.34-0.89) (**Table 2** and **Supplementary Figure S8**). SUCRA results showed that paromomycin (95.4%) was the most common treatment, followed by pentavalent antimony (52%), miltefosine (47.8%), and amphotericin B (4.7%). Rank probability plots and rank cumulative probability plots for all drugs are shown in **Figure 3F** and **Supplementary Figure S16**.

### Incidence of adverse effects-injection site pain

Of the 12 included studies, 3 studies evaluated the incidence of injection-site pain in 1357 patients, of which 307 patients experienced injection-site pain, involving 4 interventions (pentavalent antimony, amphotericin B, miltefosine, and paromomycin). The NMA further identified direct and indirect comparative analyses of these 4 interventions, and the network evidence graph is shown in **Figure 2E**. NMA results showed that the incidence of Injection site pain in the miltefosine group was lower than that in the paromomycin group (RR 0.00; 95% CI 0.00-0.01). The incidence of Injection site pain of amphotericin B was lower than that of paromomycin (RR 0.00; 95% CI 0.00-0.04). The incidence of Injection site pain in pentavalent antimony was lower than that in paromomycin (RR 0.01; 95% CI 0.00-0.14) (see **Table 2**; **Supplementary Figure S7**). The SUCRA results ranked interventions according to their probability of Injection site pain, with paromomycin (100%) ranking first, followed by pentavalent antimony (55.1%), amphotericin B (40%), and miltefosine (4.9%). Rank probability plots and rank cumulative probability plots for all drugs are shown in **Figure 3E** and **Supplementary Figure S15**.

### Publication bias and inconsistency evaluation

Publication bias was detected by observation of the symmetry of the funnel plot. The funnel plot of this study was symmetrical distribution, and all the results were not found to have obvious publication bias, with one point distributed outside the 95%CI, indicating the possible influence of a small sample size (see **Supplementary Figures S19-S26**).

## Discussion

Leishmaniasis remains a public health problem worldwide, and drug-related side effects can further limit patient adherence, but there are currently no additional drug options, this NMA was designed to evaluate the efficacy and safety of five drugs for the treatment of leishmaniasis. A random-effects model was used to analyze the NMA of clinical cure rate, mortality rate and adverse reactions of the 12 included studies, and a subgroup analysis of the efficacy of drugs in different countries was performed. To the best of our knowledge, this is the first study to analyze the efficacy of a single agent in leishmaniasis patients by NMA.

Pentavalent antimony has been used for the treatment of leishmaniasis since the 1940s and was initially considered the first-line drug in most leishmaniasis-endemic areas (Sundar and Rai, 2002). Together with amphotericin B, pentamidine, miltefosine, and paromomycin, it constitutes an option available for the chemotherapy of leishmaniasis (Garza-Tovar et al., 2020). However, it is associated with the risk of cardiotoxicity, liver cirrhosis, gastrointestinal reactions, and drug resistance (Mansuri et al., 2020; Kumari et al., 2021). The increase in the number of treatment failures in India from the 1980s has led to the necessity of a slow increase in the dose required for clinical cure (Thakur, 1984; Uliana et al., 2018). In 2010, the WHO recommended downgrading the use of pentavalent antimonial as a single agent to the second or lower option for VL treatment in all endemic regions (Organization, 2010; Uliana et al., 2018; Mokni, 2019). Amphotericin B, a macrolide polyene antibiotic, is considered a second-line treatment for CL with potent antifungal and antileishmanial activity. Its mechanism of action is that it can bind to the ergosterol of protozoan cells, causing changes in membrane integrity, leading to ion leakage and eventually cell death (Modabber, 2010; Kumari et al., 2022). Lack of response in patients to amphotericin B treatment has been reported in India, and the drug has become the first-line choice in areas where antimony is refractory widely (Kaye and Aebischer, 2011; Purkait et al., 2012; Purkait et al., 2015). The NMA results showed that the clinical cure rate of pentavalent antimony was inferior to that of amphotericin B (RR 0.23; 95% CI 0.04-1.39). The clinical cure rate of miltefosine was inferior to that of amphotericin B (RR 0.31; 95% CI 0.07-11.48). The clinical cure rate of paromomycin was inferior to that of amphotericin B (RR 0.12; 95%CI 0.01-1.55). The drugs were ranked according to their probability of clinical success. SUCRA results showed that amphotericin B had the highest cure rate, followed by miltefosine, pentavalent antimony, ketoconazole, and paromomycin. The SUCRA value is a method used to evaluate the effectiveness of different interventions, with higher SUCRA values indicating better treatment effects. In addition, the 12 studies included in this NMA were mainly from India (75%), with the remainder from Ethiopia (16.7%) and Brazil (8.3%). Subgroup analysis of pentavalent antimony efficacy in the three countries showed that Brazil (89.8%) had the highest probability of ranking first in pentavalent antimony treatment efficacy, followed by Ethiopia (33.1%) and India (27%). The low cure rate of pentavalent antimony in India may be closely related to drug resistance and other reasons. Amphotericin B may be a better choice for the treatment of leishmaniasis in India (Burza et al., 2018).

Severe side effects are the main reasons affecting the choice of medication. In the NMA analysis of drug therapy mortality, no statistical differences were found in the direct and indirect comparative efficacy of the 4 interventions (pentavalent antimony, amphotericin B, miltefosine, and paromomycin). The results of SUCRA values indicated the highest mortality rate with pentavalent antimony treatment, followed by paromomycin, amphotericin, and miltefosine. However, mortality may have been affected by limitations in treatment and diagnostic delays, as well as delays in receiving care when adverse events occur in resource-limited Settings; further studies are needed to confirm these findings.

Diarrhea is a strong predictor of mortality. Werneck et al. suggested that the occurrence of melena may be misinterpreted as diarrhea or gut microbes and may be related to sepsis associated with abnormal coagulation (Werneck et al., 2003). In the analysis of the incidence of adverse effects in patients with leishmaniasis, we found that vomiting and diarrhea were most common in patients treated with miltefosine, and pain at the injection site and abnormalities in aspartate aminotransferase and alanine aminotransferase were most common in patients treated with paromomycin. Miltefosine was initially developed as an anticancer agent but was reported for the treatment of leishmaniasis after studies found that its toxicity to the kidney and GI tract could not be avoided and became an important alternative for the treatment of Post-kala-azar dermal leishmaniasis (PKDL) in India (Croft and Engel, 2006; Sundar et al., 2006; Sundar and Olliaro, 2007). Joyce et al. found that adverse effects of miltefosine occurred in the first week of treatment and may become more severe after 4 weeks of treatment; most of these side effects were related to the gastrointestinal tract, such as diarrhea, nausea, and vomiting, which are consistent with the results of our study (Pijpers et al., 2019). Dorlo et al. found similar side effects in their review of miltefosine for the treatment of VL, possibly because miltefosine acts on the gastrointestinal mucosa (Dorlo et al., 2012). Paromomycin is an antibiotic agent that emerged as a new candidate for the treatment of visceral leishmaniasis in 2006 (Mondal et al., 2010). Paromomycin kills Leishmania protozoa in a complex manner and can kill Leishmania dubliniensis by inhibiting parasite metabolism and mitochondrial respiration (Maarouf et al., 1997). It is usually administered by intramuscular injection, which predisposes patients to pain at the injection site. However, liver enzyme abnormalities were more common in patients receiving paromomycin (Sundar et al., 2007; Sinha et al., 2011). Shyam Sundar et al. found that liver function test values peaked after the start of paromomycin treatment and then decreased to baseline at end of treatment (EOT) (Sundar et al., 2007). The liver is an important site of drug metabolism in the human body, which can convert drugs into more easily excreted forms through various enzyme systems. Paromomycin has a greater effect on the liver after treatment, which may be related to its complex chemical structure or its metabolic pathway.

The study also has some limitations: First, some of the included studies had open-label studies, but this may not have affected the outcome measures because these were determined by objective criteria. Secondly, there are differences in the dose of amphotericin B treatment among the studies, which are not completely consistent. Thirdly, due to the lack of intersection of different types of leishmaniasis therapeutic drugs and the small proportion of CL, subgroup analysis of different types of leishmaniasis therapeutic drugs was not performed in this study. Finally, most of the studies included in this NMA were from India, and it is necessary to be cautious in generalizing this result to other endemic countries, and more high-quality multi-center clinical studies are needed in the future.

## Conclusion

The results of this systematic review and NMA are the first analysis of drugs for the treatment of leishmaniasis. The results indicate that amphotericin B may be the most effective choice for the treatment of leishmaniasis. Miltefosine, pentavalent antimony, and paromomycin all have varying degrees of adverse reactions in the treatment of leishmaniasis.

## Data Availability

The datasets presented in this study can be found in online repositories. The names of the repository/repositories and accession number(s) can be found in the article/**Supplementary Material**.
