# Supplementary material for: Efficacy and safety of different drugs for the treatment of leishmaniasis: a systematic review and network meta-analysis
Source: Front Cell Infect Microbiol. 2025 Dec 15;15:1717863. doi: 10.3389/fcimb.2025.1717863 (PMC12745438; doi:10.3389/fcimb.2025.1717863)
Supplement: Supplementary file 1 [file Supplementaryfile1.docx]

**Efficacy and safety of different drugs for the treatment of leishmaniasis: a systematic review and network meta-analysis**

**Content list**

**eTable 1 PubMed search strategy and result**

**eTable 2 Embase search strategy and result**

**eTable 3 Cochrane search strategy and result**

**eFigure 1 Risk of bias summary**

**eFigure 2 Risk of bias graph**

**eFigure 3 Forest plot of the network meta-analysis of clinical cure rates**

**eFigure 4 Forest plot of the network meta-analysis of mortality**

**eFigure 5 Forest plot of network meta-analysis of adverse effects (Vomiting)**

**eFigure 6 Forest plot of network meta-analysis of adverse effects (Diarrhea)**

**eFigure 7 Forest plot of network meta-analysis of adverse effects (Injection site pain)**

**eFigure 8 Forest plot of network meta-analysis of adverse effects (Abnormal alanine aminotransferase)**

**eFigure 9 Forest plot of network meta-analysis of adverse effects (Abnormal aspartate aminotransferase)**

**eFigure 10 Forest map of the efficacy of pentavalent antimony in treating leishmaniasis in different countries**

**eFigure 11 SUCRA efficacy ranking curve is clinical cure rate, SUCRA = cumulative ranking surface**

**eFigure 12 SUCRA efficacy ranking curve is mortality, SUCRA = cumulative ranking surface**

**eFigure 13 SUCRA efficacy ranking curve is the incidence of adverse reactions (Vomiting), SUCRA = cumulative ranking surface**

**eFigure 14 SUCRA efficacy ranking curve is the incidence of adverse reactions (Diarrhea), SUCRA = cumulative ranking surface**

**eFigure 15 SUCRA efficacy ranking curve is the incidence of adverse reactions (Injection site pain), SUCRA = cumulative ranking surface**

**eFigure 16 SUCRA efficacy ranking curve is the incidence of adverse reactions (Abnormal alanine aminotransferase), SUCRA = cumulative ranking surface**

**eFigure 17 SUCRA efficacy ranking curve is the incidence of adverse reactions (Abnormal aspartate aminotransferase), SUCRA = cumulative ranking surface**

**eFigure 18 SUCRA efficacy ranking curve is the clinical cure rate of leishmaniasis treated with pentavalent antimony in different countries, SUCRA = cumulative ranking surface**

**eFigure 19 Funnel plot of a network meta-analysis of clinical cure rates**

**eFigure 20 Funnel plot of a network meta-analysis of mortality**

**eFigure 21 Funnel plot of a network meta-analysis of the incidence of adverse events（Vomiting）**

**eFigure 22 Funnel plot of a network meta-analysis of the incidence of adverse events（Diarrhea）**

**eFigure 23 Funnel plot of a network meta-analysis of the incidence of adverse events（Injection site pain）**

**eFigure 24 Funnel plot of a network meta-analysis of the incidence of adverse events（Abnormal alanine aminotransferase）**

**eFigure 25 Funnel plot of a network meta-analysis of the incidence of adverse events（Abnormal aspartate aminotransferase）**

**eFigure 26 Funnel plot of a network meta-analysis of the clinical cure rate of leishmaniasis treated with pentavalent antimony in different countries**

**eTable 1 PubMed search strategy and result**

| Search number | Query | Results | Time |
| --- | --- | --- | --- |
| 1 | "Leishmaniasis, Visceral"[Mesh] | 11,197 | 6:34:14 |
| 2 | ((((Visceral Leishmaniasis[Title/Abstract]) OR (Black Fever[Title/Abstract])) OR (Fever, Black[Title/Abstract])) OR (Kala-Azar[Title/Abstract])) OR (Kala Azar[Title/Abstract]) | 11,725 | 6:36:00 |
| 3 | (((((Visceral Leishmaniasis[Title/Abstract]) OR (Black Fever[Title/Abstract])) OR (Fever, Black[Title/Abstract])) OR (Kala-Azar[Title/Abstract])) OR (Kala Azar[Title/Abstract])) OR ("Leishmaniasis, Visceral"[Mesh]) | 14,321 | 6:36:45 |
| 4 | "Amphotericin B"[Mesh] | 16,587 | 6:37:31 |
| 5 | ((((Amphotericin[Title/Abstract]) OR (Fungizone[Title/Abstract])) OR (Amphotericin B Cholesterol Dispersion[Title/Abstract])) OR (Amphotericin B Colloidal Dispersion[Title/Abstract])) OR (Amphocil[Title/Abstract]) | 22,777 | 6:38:31 |
| 6 | (((((Amphotericin[Title/Abstract]) OR (Fungizone[Title/Abstract])) OR (Amphotericin B Cholesterol Dispersion[Title/Abstract])) OR (Amphotericin B Colloidal Dispersion[Title/Abstract])) OR (Amphocil[Title/Abstract])) OR ("Amphotericin B"[Mesh]) | 27,444 | 6:38:53 |
| 7 | "miltefosine" [Supplementary Concept] | 1,086 | 6:43:15 |
| 8 | (((((((n-hexadecylphosphorylcholine[Title/Abstract]) OR (HDPC[Title/Abstract])) OR (hexadecylphosphocholine[Title/Abstract])) OR (Miltex[Title/Abstract])) OR (Impavido[Title/Abstract])) OR (D 18506[Title/Abstract])) OR (D18506[Title/Abstract])) OR (D-18506[Title/Abstract]) | 483 | 6:44:09 |
| 9 | ("miltefosine" [Supplementary Concept]) OR ((((((((n-hexadecylphosphorylcholine[Title/Abstract]) OR (HDPC[Title/Abstract])) OR (hexadecylphosphocholine[Title/Abstract])) OR (Miltex[Title/Abstract])) OR (Impavido[Title/Abstract])) OR (D 18506[Title/Abstract])) OR (D18506[Title/Abstract])) OR (D-18506[Title/Abstract])) | 1,329 | 6:44:25 |
| 10 | "Antimony Sodium Gluconate"[Mesh] | 920 | 6:44:54 |
| 11 | ((((((((((((Sodium Stibogluconate[Title/Abstract]) OR (Stibogluconate, Sodium[Title/Abstract])) OR (Stibogluconate Sodium[Title/Abstract])) OR (Sodium, Stibogluconate[Title/Abstract])) OR (Antimony Gluconate Sodium[Title/Abstract])) OR (Antimony Sodium Gluconates[Title/Abstract])) OR (Sodium Gluconates, Antimony[Title/Abstract])) OR (Myostibin[Title/Abstract])) OR (Triostam[Title/Abstract])) OR (Solustibosan[Title/Abstract])) OR (Stibatin[Title/Abstract])) OR (Antimony Gluconic Acid[Title/Abstract])) OR (Pentostam[Title/Abstract]) | 1,102 | 6:46:09 |
| 12 | ("Antimony Sodium Gluconate"[Mesh]) OR (((((((((((((Sodium Stibogluconate[Title/Abstract]) OR (Stibogluconate, Sodium[Title/Abstract])) OR (Stibogluconate Sodium[Title/Abstract])) OR (Sodium, Stibogluconate[Title/Abstract])) OR (Antimony Gluconate Sodium[Title/Abstract])) OR (Antimony Sodium Gluconates[Title/Abstract])) OR (Sodium Gluconates, Antimony[Title/Abstract])) OR (Myostibin[Title/Abstract])) OR (Triostam[Title/Abstract])) OR (Solustibosan[Title/Abstract])) OR (Stibatin[Title/Abstract])) OR (Antimony Gluconic Acid[Title/Abstract])) OR (Pentostam[Title/Abstract])) | 1,364 | 6:46:24 |
| 13 | "Paromomycin"[Mesh] | 1,385 | 6:53:44 |
| 14 | (((((((((((((((Estomycin[Title/Abstract]) OR (Aminosidine[Title/Abstract])) OR (Neomycin E[Title/Abstract])) OR (Paramomycin[Title/Abstract])) OR (Paromomycin I[Title/Abstract])) OR (Catenulin[Title/Abstract])) OR (Hydroxymycin[Title/Abstract])) OR (Paromomycin Sulfate (1:1[Title/Abstract]))) OR (Paromomycin Sulfate (2:5[Title/Abstract]))) OR (Paromomycin Phosphate[Title/Abstract])) OR (Paromomycin, beta-D-Glucopyranosyl-Isomer[Title/Abstract])) OR (Paromomycin, beta D Glucopyranosyl Isomer[Title/Abstract])) OR (beta-D-Glucopyranosyl-Isomer Paromomycin[Title/Abstract])) OR (Humatin[Title/Abstract])) OR (Paromomycin Sulfate[Title/Abstract])) OR (Gabbromycin[Title/Abstract]) | 1,720 | 6:56:11 |
| 15 | ("Paromomycin"[Mesh]) OR ((((((((((((((((Estomycin[Title/Abstract]) OR (Aminosidine[Title/Abstract])) OR (Neomycin E[Title/Abstract])) OR (Paramomycin[Title/Abstract])) OR (Paromomycin I[Title/Abstract])) OR (Catenulin[Title/Abstract])) OR (Hydroxymycin[Title/Abstract])) OR (Paromomycin Sulfate (1:1[Title/Abstract]))) OR (Paromomycin Sulfate (2:5[Title/Abstract]))) OR (Paromomycin Phosphate[Title/Abstract])) OR (Paromomycin, beta-D-Glucopyranosyl-Isomer[Title/Abstract])) OR (Paromomycin, beta D Glucopyranosyl Isomer[Title/Abstract])) OR (beta-D-Glucopyranosyl-Isomer Paromomycin[Title/Abstract])) OR (Humatin[Title/Abstract])) OR (Paromomycin Sulfate[Title/Abstract])) OR (Gabbromycin[Title/Abstract])) | 2,144 | 6:56:26 |
| 16 | (Randomized controlled trial[Publication Type]) OR (randomized[Title/Abstract]) | 967,531 | 6:57:21 |
| 17 | ((((((Visceral Leishmaniasis[Title/Abstract]) OR (Black Fever[Title/Abstract])) OR (Fever, Black[Title/Abstract])) OR (Kala-Azar[Title/Abstract])) OR (Kala Azar[Title/Abstract])) OR ("Leishmaniasis, Visceral"[Mesh])) AND ((Randomized controlled trial[Publication Type]) OR (randomized[Title/Abstract])) | 190 | 6:57:46 |
| 18 | ((((((Amphotericin[Title/Abstract]) OR (Fungizone[Title/Abstract])) OR (Amphotericin B Cholesterol Dispersion[Title/Abstract])) OR (Amphotericin B Colloidal Dispersion[Title/Abstract])) OR (Amphocil[Title/Abstract])) OR ("Amphotericin B"[Mesh])) AND ((Randomized controlled trial[Publication Type]) OR (randomized[Title/Abstract])) | 790 | 6:58:09 |
| 19 | (("miltefosine" [Supplementary Concept]) OR ((((((((n-hexadecylphosphorylcholine[Title/Abstract]) OR (HDPC[Title/Abstract])) OR (hexadecylphosphocholine[Title/Abstract])) OR (Miltex[Title/Abstract])) OR (Impavido[Title/Abstract])) OR (D 18506[Title/Abstract])) OR (D18506[Title/Abstract])) OR (D-18506[Title/Abstract]))) AND ((Randomized controlled trial[Publication Type]) OR (randomized[Title/Abstract])) | 56 | 6:58:27 |
| 20 | (("Antimony Sodium Gluconate"[Mesh]) OR (((((((((((((Sodium Stibogluconate[Title/Abstract]) OR (Stibogluconate, Sodium[Title/Abstract])) OR (Stibogluconate Sodium[Title/Abstract])) OR (Sodium, Stibogluconate[Title/Abstract])) OR (Antimony Gluconate Sodium[Title/Abstract])) OR (Antimony Sodium Gluconates[Title/Abstract])) OR (Sodium Gluconates, Antimony[Title/Abstract])) OR (Myostibin[Title/Abstract])) OR (Triostam[Title/Abstract])) OR (Solustibosan[Title/Abstract])) OR (Stibatin[Title/Abstract])) OR (Antimony Gluconic Acid[Title/Abstract])) OR (Pentostam[Title/Abstract]))) AND ((Randomized controlled trial[Publication Type]) OR (randomized[Title/Abstract])) | 75 | 6:58:38 |
| 21 | (("Paromomycin"[Mesh]) OR ((((((((((((((((Estomycin[Title/Abstract]) OR (Aminosidine[Title/Abstract])) OR (Neomycin E[Title/Abstract])) OR (Paramomycin[Title/Abstract])) OR (Paromomycin I[Title/Abstract])) OR (Catenulin[Title/Abstract])) OR (Hydroxymycin[Title/Abstract])) OR (Paromomycin Sulfate (1:1[Title/Abstract]))) OR (Paromomycin Sulfate (2:5[Title/Abstract]))) OR (Paromomycin Phosphate[Title/Abstract])) OR (Paromomycin, beta-D-Glucopyranosyl-Isomer[Title/Abstract])) OR (Paromomycin, beta D Glucopyranosyl Isomer[Title/Abstract])) OR (beta-D-Glucopyranosyl-Isomer Paromomycin[Title/Abstract])) OR (Humatin[Title/Abstract])) OR (Paromomycin Sulfate[Title/Abstract])) OR (Gabbromycin[Title/Abstract]))) AND ((Randomized controlled trial[Publication Type]) OR (randomized[Title/Abstract])) | 81 | 6:58:53 |
| 22 | (((((((Visceral Leishmaniasis[Title/Abstract]) OR (Black Fever[Title/Abstract])) OR (Fever, Black[Title/Abstract])) OR (Kala-Azar[Title/Abstract])) OR (Kala Azar[Title/Abstract])) OR ("Leishmaniasis, Visceral"[Mesh])) AND ((Randomized controlled trial[Publication Type]) OR (randomized[Title/Abstract]))) AND (((((((Amphotericin[Title/Abstract]) OR (Fungizone[Title/Abstract])) OR (Amphotericin B Cholesterol Dispersion[Title/Abstract])) OR (Amphotericin B Colloidal Dispersion[Title/Abstract])) OR (Amphocil[Title/Abstract])) OR ("Amphotericin B"[Mesh])) AND ((Randomized controlled trial[Publication Type]) OR (randomized[Title/Abstract]))) | 61 | 6:59:52 |
| 23 | (((((((Visceral Leishmaniasis[Title/Abstract]) OR (Black Fever[Title/Abstract])) OR (Fever, Black[Title/Abstract])) OR (Kala-Azar[Title/Abstract])) OR (Kala Azar[Title/Abstract])) OR ("Leishmaniasis, Visceral"[Mesh])) AND ((Randomized controlled trial[Publication Type]) OR (randomized[Title/Abstract]))) AND ((("miltefosine" [Supplementary Concept]) OR ((((((((n-hexadecylphosphorylcholine[Title/Abstract]) OR (HDPC[Title/Abstract])) OR (hexadecylphosphocholine[Title/Abstract])) OR (Miltex[Title/Abstract])) OR (Impavido[Title/Abstract])) OR (D 18506[Title/Abstract])) OR (D18506[Title/Abstract])) OR (D-18506[Title/Abstract]))) AND ((Randomized controlled trial[Publication Type]) OR (randomized[Title/Abstract]))) | 28 | 7:00:10 |
| 24 | (((((((Visceral Leishmaniasis[Title/Abstract]) OR (Black Fever[Title/Abstract])) OR (Fever, Black[Title/Abstract])) OR (Kala-Azar[Title/Abstract])) OR (Kala Azar[Title/Abstract])) OR ("Leishmaniasis, Visceral"[Mesh])) AND ((Randomized controlled trial[Publication Type]) OR (randomized[Title/Abstract]))) AND ((("Antimony Sodium Gluconate"[Mesh]) OR (((((((((((((Sodium Stibogluconate[Title/Abstract]) OR (Stibogluconate, Sodium[Title/Abstract])) OR (Stibogluconate Sodium[Title/Abstract])) OR (Sodium, Stibogluconate[Title/Abstract])) OR (Antimony Gluconate Sodium[Title/Abstract])) OR (Antimony Sodium Gluconates[Title/Abstract])) OR (Sodium Gluconates, Antimony[Title/Abstract])) OR (Myostibin[Title/Abstract])) OR (Triostam[Title/Abstract])) OR (Solustibosan[Title/Abstract])) OR (Stibatin[Title/Abstract])) OR (Antimony Gluconic Acid[Title/Abstract])) OR (Pentostam[Title/Abstract]))) AND ((Randomized controlled trial[Publication Type]) OR (randomized[Title/Abstract]))) | 41 | 7:00:27 |
| 25 | (((((((Visceral Leishmaniasis[Title/Abstract]) OR (Black Fever[Title/Abstract])) OR (Fever, Black[Title/Abstract])) OR (Kala-Azar[Title/Abstract])) OR (Kala Azar[Title/Abstract])) OR ("Leishmaniasis, Visceral"[Mesh])) AND ((Randomized controlled trial[Publication Type]) OR (randomized[Title/Abstract]))) AND ((("Paromomycin"[Mesh]) OR ((((((((((((((((Estomycin[Title/Abstract]) OR (Aminosidine[Title/Abstract])) OR (Neomycin E[Title/Abstract])) OR (Paramomycin[Title/Abstract])) OR (Paromomycin I[Title/Abstract])) OR (Catenulin[Title/Abstract])) OR (Hydroxymycin[Title/Abstract])) OR (Paromomycin Sulfate (1:1[Title/Abstract]))) OR (Paromomycin Sulfate (2:5[Title/Abstract]))) OR (Paromomycin Phosphate[Title/Abstract])) OR (Paromomycin, beta-D-Glucopyranosyl-Isomer[Title/Abstract])) OR (Paromomycin, beta D Glucopyranosyl Isomer[Title/Abstract])) OR (beta-D-Glucopyranosyl-Isomer Paromomycin[Title/Abstract])) OR (Humatin[Title/Abstract])) OR (Paromomycin Sulfate[Title/Abstract])) OR (Gabbromycin[Title/Abstract]))) AND ((Randomized controlled trial[Publication Type]) OR (randomized[Title/Abstract]))) | 28 | 7:00:41 |
| 26 | ((((((((((Visceral Leishmaniasis[Title/Abstract]) OR (Black Fever[Title/Abstract])) OR (Fever, Black[Title/Abstract])) OR (Kala-Azar[Title/Abstract])) OR (Kala Azar[Title/Abstract])) OR ("Leishmaniasis, Visceral"[Mesh])) AND ((Randomized controlled trial[Publication Type]) OR (randomized[Title/Abstract]))) AND (((((((Amphotericin[Title/Abstract]) OR (Fungizone[Title/Abstract])) OR (Amphotericin B Cholesterol Dispersion[Title/Abstract])) OR (Amphotericin B Colloidal Dispersion[Title/Abstract])) OR (Amphocil[Title/Abstract])) OR ("Amphotericin B"[Mesh])) AND ((Randomized controlled trial[Publication Type]) OR (randomized[Title/Abstract])))) OR ((((((((Visceral Leishmaniasis[Title/Abstract]) OR (Black Fever[Title/Abstract])) OR (Fever, Black[Title/Abstract])) OR (Kala-Azar[Title/Abstract])) OR (Kala Azar[Title/Abstract])) OR ("Leishmaniasis, Visceral"[Mesh])) AND ((Randomized controlled trial[Publication Type]) OR (randomized[Title/Abstract]))) AND ((("miltefosine" [Supplementary Concept]) OR ((((((((n-hexadecylphosphorylcholine[Title/Abstract]) OR (HDPC[Title/Abstract])) OR (hexadecylphosphocholine[Title/Abstract])) OR (Miltex[Title/Abstract])) OR (Impavido[Title/Abstract])) OR (D 18506[Title/Abstract])) OR (D18506[Title/Abstract])) OR (D-18506[Title/Abstract]))) AND ((Randomized controlled trial[Publication Type]) OR (randomized[Title/Abstract]))))) OR ((((((((Visceral Leishmaniasis[Title/Abstract]) OR (Black Fever[Title/Abstract])) OR (Fever, Black[Title/Abstract])) OR (Kala-Azar[Title/Abstract])) OR (Kala Azar[Title/Abstract])) OR ("Leishmaniasis, Visceral"[Mesh])) AND ((Randomized controlled trial[Publication Type]) OR (randomized[Title/Abstract]))) AND ((("Antimony Sodium Gluconate"[Mesh]) OR (((((((((((((Sodium Stibogluconate[Title/Abstract]) OR (Stibogluconate, Sodium[Title/Abstract])) OR (Stibogluconate Sodium[Title/Abstract])) OR (Sodium, Stibogluconate[Title/Abstract])) OR (Antimony Gluconate Sodium[Title/Abstract])) OR (Antimony Sodium Gluconates[Title/Abstract])) OR (Sodium Gluconates, Antimony[Title/Abstract])) OR (Myostibin[Title/Abstract])) OR (Triostam[Title/Abstract])) OR (Solustibosan[Title/Abstract])) OR (Stibatin[Title/Abstract])) OR (Antimony Gluconic Acid[Title/Abstract])) OR (Pentostam[Title/Abstract]))) AND ((Randomized controlled trial[Publication Type]) OR (randomized[Title/Abstract]))))) OR ((((((((Visceral Leishmaniasis[Title/Abstract]) OR (Black Fever[Title/Abstract])) OR (Fever, Black[Title/Abstract])) OR (Kala-Azar[Title/Abstract])) OR (Kala Azar[Title/Abstract])) OR ("Leishmaniasis, Visceral"[Mesh])) AND ((Randomized controlled trial[Publication Type]) OR (randomized[Title/Abstract]))) AND ((("Paromomycin"[Mesh]) OR ((((((((((((((((Estomycin[Title/Abstract]) OR (Aminosidine[Title/Abstract])) OR (Neomycin E[Title/Abstract])) OR (Paramomycin[Title/Abstract])) OR (Paromomycin I[Title/Abstract])) OR (Catenulin[Title/Abstract])) OR (Hydroxymycin[Title/Abstract])) OR (Paromomycin Sulfate (1:1[Title/Abstract]))) OR (Paromomycin Sulfate (2:5[Title/Abstract]))) OR (Paromomycin Phosphate[Title/Abstract])) OR (Paromomycin, beta-D-Glucopyranosyl-Isomer[Title/Abstract])) OR (Paromomycin, beta D Glucopyranosyl Isomer[Title/Abstract])) OR (beta-D-Glucopyranosyl-Isomer Paromomycin[Title/Abstract])) OR (Humatin[Title/Abstract])) OR (Paromomycin Sulfate[Title/Abstract])) OR (Gabbromycin[Title/Abstract]))) AND ((Randomized controlled trial[Publication Type]) OR (randomized[Title/Abstract])))) | 106 | 7:04:37 |

**eTable 2 Embase search strategy and result**

| No. | Query | Results | Date |
| --- | --- | --- | --- |
| #1 | 'kala-azar':ab,ti | 2931 | 20-Feb-24 |
| #2 | 'visceral leishmaniasis':ab,ti | 11239 | 20-Feb-24 |
| #3 | 'black fever':ab,ti | 24 | 20-Feb-24 |
| #4 | 'fever, black':ab,ti | 5 | 20-Feb-24 |
| #5 | 'kala-azar':ab,ti | 2931 | 20-Feb-24 |
| #6 | 'kala azar':ab,ti | 2931 | 20-Feb-24 |
| #7 | #1 OR #2 OR #3 OR #4 OR #5 OR #6 | 12909 | 20-Feb-24 |
| #8 | 'amphotericin b':ti,ab | 27140 | 20-Feb-24 |
| #9 | 'amphotericin':ti,ab | 29077 | 20-Feb-24 |
| #10 | 'fungizone':ti,ab | 449 | 20-Feb-24 |
| #11 | 'amphotericin b cholesterol dispersion':ti,ab | 3 | 20-Feb-24 |
| #12 | 'amphotericin b colloidal dispersion':ti,ab | 131 | 20-Feb-24 |
| #13 | 'amphocil':ti,ab | 52 | 20-Feb-24 |
| #14 | #8 OR #9 OR #10 OR #11 OR #12 OR #13 | 29184 | 20-Feb-24 |
| #15 | 'miltefosine':ab,ti | 1892 | 20-Feb-24 |
| #16 | 'n-hexadecylphosphorylcholine':ab,ti | 4 | 20-Feb-24 |
| #17 | 'hdpc':ab,ti | 179 | 20-Feb-24 |
| #18 | 'hexadecylphosphocholine':ab,ti | 339 | 20-Feb-24 |
| #19 | 'miltex':ab,ti | 31 | 20-Feb-24 |
| #20 | 'impavido':ab,ti | 22 | 20-Feb-24 |
| #21 | 'd 18506':ab,ti | 9 | 20-Feb-24 |
| #22 | 'd18506':ab,ti | 7 | 20-Feb-24 |
| #23 | 'd-18506':ab,ti | 9 | 20-Feb-24 |
| #24 | #15 OR #16 OR #17 OR #18 OR #19 OR #20 OR #21 OR #22 OR #23 | 2298 | 20-Feb-24 |
| #25 | 'antimony sodium gluconate':ab,ti | 6 | 20-Feb-24 |
| #26 | 'sodium stibogluconate':ab,ti | 849 | 20-Feb-24 |
| #27 | 'stibogluconate, sodium':ab,ti | 7 | 20-Feb-24 |
| #28 | 'stibogluconate sodium':ab,ti | 7 | 20-Feb-24 |
| #29 | 'sodium, stibogluconate':ab,ti | 849 | 20-Feb-24 |
| #30 | 'antimony gluconate sodium':ab,ti | 1 | 20-Feb-24 |
| #31 | 'antimony sodium gluconates':ab,ti | 0 | 20-Feb-24 |
| #32 | 'sodium gluconates, antimony':ab,ti | 0 | 20-Feb-24 |
| #33 | 'myostibin':ab,ti | 0 | 20-Feb-24 |
| #34 | 'triostam':ab,ti | 13 | 20-Feb-24 |
| #35 | 'solustibosan':ab,ti | 4 | 20-Feb-24 |
| #36 | 'stibatin':ab,ti | 2 | 20-Feb-24 |
| #37 | 'antimony gluconic acid':ab,ti | 0 | 20-Feb-24 |
| #38 | 'pentostam':ab,ti | 272 | 20-Feb-24 |
| #39 | #25 OR #26 OR #27 OR #28 OR #29 OR #30 OR #31 OR #32 OR #33 OR #34 OR #35 OR #36 OR #37 OR #38 | 1041 | 20-Feb-24 |
| #40 | 'paromomycin':ab,ti | 1685 | 20-Feb-24 |
| #41 | 'estomycin':ab,ti | 0 | 20-Feb-24 |
| #42 | 'aminosidine':ab,ti | 160 | 20-Feb-24 |
| #43 | 'neomycin e':ab,ti | 6 | 20-Feb-24 |
| #44 | 'paramomycin':ab,ti | 78 | 20-Feb-24 |
| #45 | 'paromomycin i':ab,ti | 11 | 20-Feb-24 |
| #46 | 'catenulin':ab,ti | 1 | 20-Feb-24 |
| #47 | 'hydroxymycin':ab,ti | 1 | 20-Feb-24 |
| #48 | 'paromomycin sulfate (1:1)':ab,ti | 0 | 20-Feb-24 |
| #49 | 'paromomycin sulfate (2:5)':ab,ti | 0 | 20-Feb-24 |
| #50 | 'paromomycin phosphate':ab,ti | 0 | 20-Feb-24 |
| #51 | 'paromomycin, beta-d-glucopyranosyl-isomer':ab,ti | 0 | 20-Feb-24 |
| #52 | 'paromomycin, beta d glucopyranosyl isomer':ab,ti | 0 | 20-Feb-24 |
| #53 | 'beta-d-glucopyranosyl-isomer paromomycin':ab,ti | 0 | 20-Feb-24 |
| #54 | 'humatin':ab,ti | 32 | 20-Feb-24 |
| #55 | 'paromomycin sulfate':ab,ti | 98 | 20-Feb-24 |
| #56 | 'gabbromycin':ab,ti | 15 | 20-Feb-24 |
| #57 | #40 OR #41 OR #42 OR #43 OR #44 OR #45 OR #46 OR #47 OR #48 OR #49 OR #50 OR #51 OR #52 OR #53 OR #54 OR #55 OR #56 | 1917 | 20-Feb-24 |
| #58 | 'random':ti,ab OR 'placebo':ti,ab OR 'double blind':ti,ab | 880752 | 20-Feb-24 |
| #59 | #14 OR #24 OR #39 OR #56 | 31767 | 20-Feb-24 |
| #60 | #7 AND #58 AND #59 | 19 | 20-Feb-24 |

**eTable 3 Cochrane search strategy and result**

| ID | Search |
| --- | --- |
| #1 | MeSH descriptor: [Leishmaniasis, Visceral] explode all trees |
| #2 | (Visceral Leishmaniasis):ti,ab,kw OR (Black Fever):ti,ab,kw OR (Fever, Black):ti,ab,kw OR (Kala-Azar):ti,ab,kw OR (Kala Azar):ti,ab,kw (Word variations have been searched) |
| #3 | #1 or #2 |
| #4 | MeSH descriptor: [Amphotericin B] explode all trees |
| #5 | (Amphotericin):ti,ab,kw OR (Fungizone):ti,ab,kw OR (Amphotericin B Cholesterol Dispersion):ti,ab,kw OR (Amphotericin B Colloidal Dispersion):ti,ab,kw OR (Amphocil):ti,ab,kw (Word variations have been searched) |
| #6 | #5 or #4 |
| #7 | MeSH descriptor: [] explode all trees |
| #8 | (n-hexadecylphosphorylcholine):ti,ab,kw OR (HDPC):ti,ab,kw OR (hexadecylphosphocholine):ti,ab,kw OR (Miltex):ti,ab,kw OR (Impavido):ti,ab,kw (Word variations have been searched) |
| #9 | (D 18506):ti,ab,kw OR (D18506):ti,ab,kw OR (D-18506):ti,ab,kw (Word variations have been searched) |
| #10 | #7 or #8 or #9 |
| #11 | MeSH descriptor: [Antimony Sodium Gluconate] explode all trees |
| #12 | (Sodium Stibogluconate):ti,ab,kw OR (Stibogluconate, Sodium):ti,ab,kw OR (Stibogluconate Sodium):ti,ab,kw OR (Sodium, Stibogluconate):ti,ab,kw OR (Antimony Gluconate Sodium):ti,ab,kw (Word variations have been searched) |
| #13 | (Antimony Sodium Gluconates):ti,ab,kw OR (Sodium Gluconates, Antimony):ti,ab,kw OR (Myostibin):ti,ab,kw OR (Triostam):ti,ab,kw OR (Solustibosan):ti,ab,kw (Word variations have been searched) |
| #14 | (Stibatin):ti,ab,kw OR (Antimony Gluconic Acid):ti,ab,kw OR (Pentostam):ti,ab,kw (Word variations have been searched) |
| #15 | #11 or #12 or #13 or #14 |
| #16 | MeSH descriptor: [Paromomycin] explode all trees |
| #17 | (Estomycin):ti,ab,kw OR (Aminosidine):ti,ab,kw OR (Neomycin E):ti,ab,kw OR (Paramomycin):ti,ab,kw OR (Paromomycin I):ti,ab,kw (Word variations have been searched) |
| #18 | (Catenulin):ti,ab,kw OR (Hydroxymycin):ti,ab,kw OR (Paromomycin Phosphate):ti,ab,kw OR (Paromomycin, beta-D-Glucopyranosyl-Isomer):ti,ab,kw OR (Paromomycin, beta D Glucopyranosyl Isomer):ti,ab,kw (Word variations have been searched) |
| #19 | (beta-D-Glucopyranosyl-Isomer Paromomycin):ti,ab,kw OR (Humatin):ti,ab,kw OR (Paromomycin Sulfate):ti,ab,kw OR (Gabbromycin):ti,ab,kw (Word variations have been searched) |
| #20 | #16 or #17 or #18 or #19 |
| #21 | #6 or #10 or #15 or #20 |
| #22 | #3 and #21 |


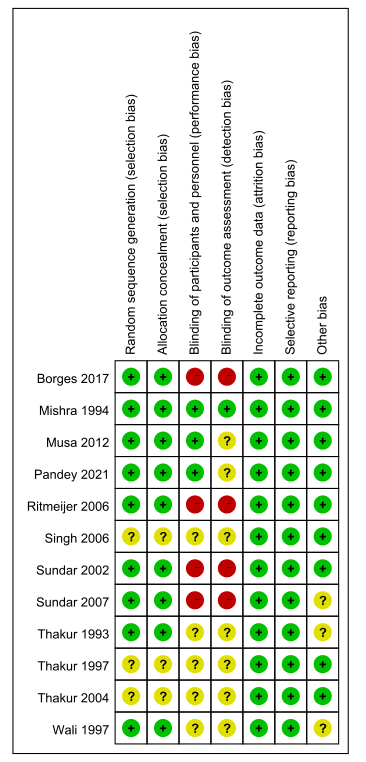


**eFigure 1 Risk of bias summary**


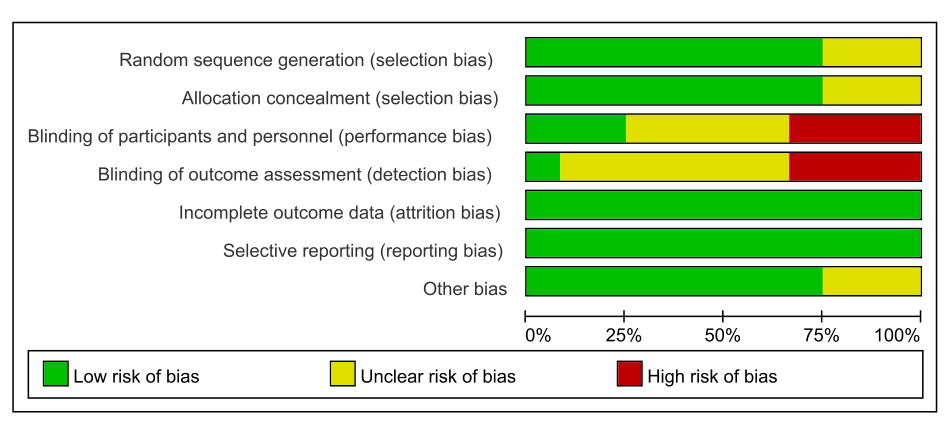


**eFigure 2 Risk of bias graph**


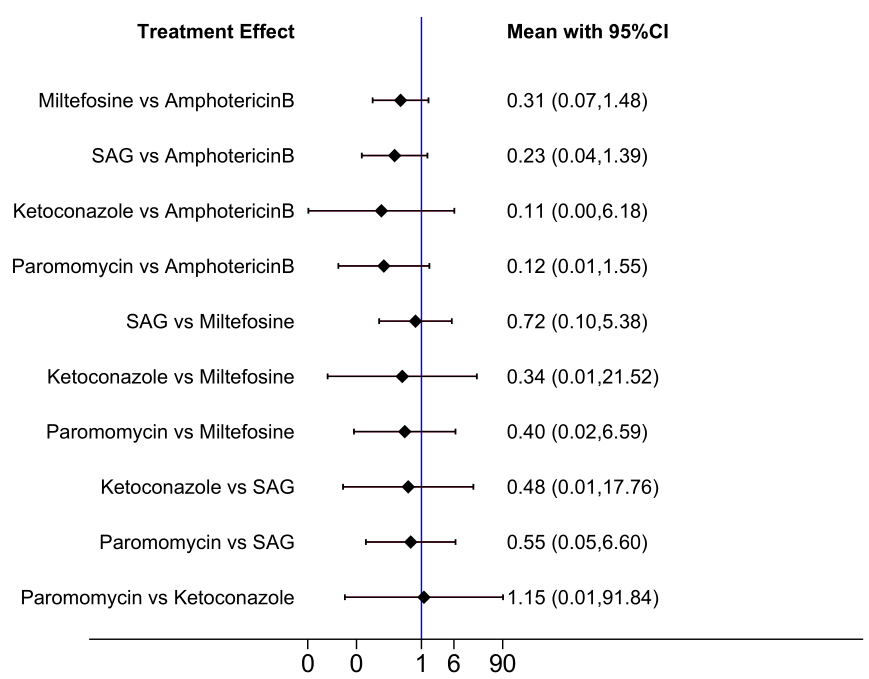


**eFigure 3 Forest plot of the network meta-analysis of clinical cure rates**


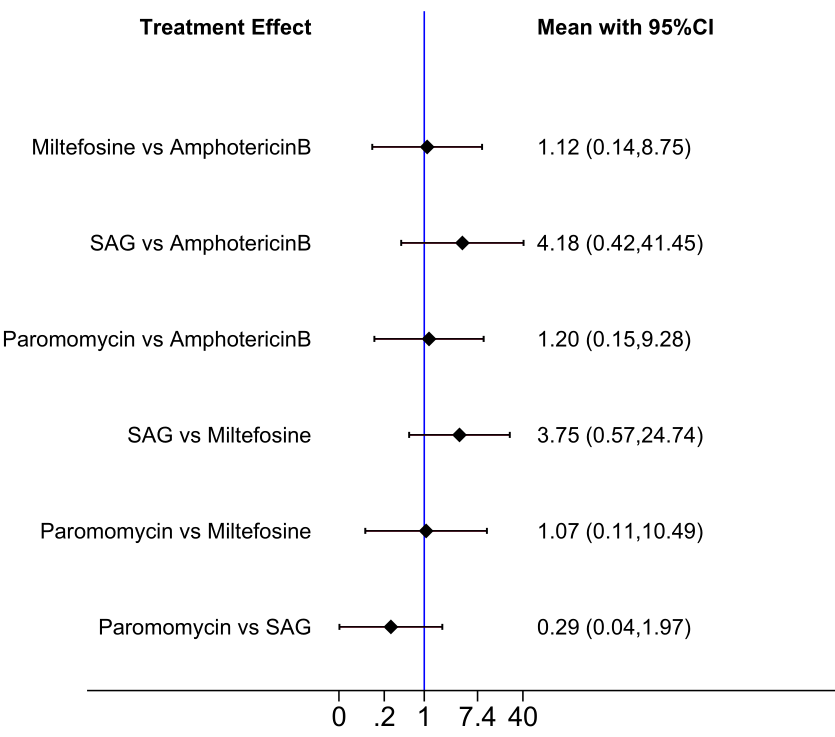


**eFigure 4 Forest plot of the network meta-analysis of mortality**


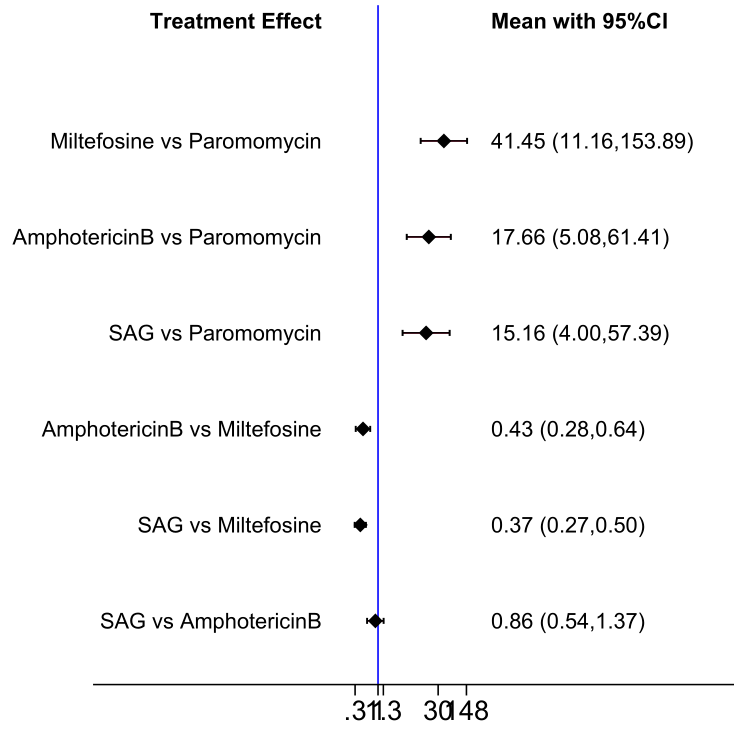


**eFigure 5 Forest plot of network meta-analysis of adverse effects (Vomiting)**


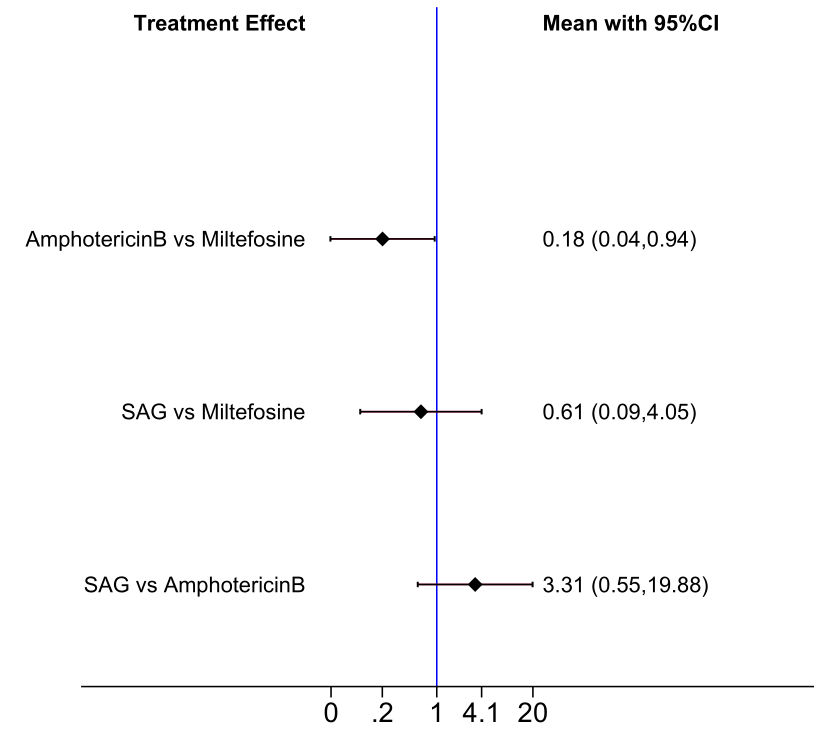


**eFigure 6 Forest plot of network meta-analysis of adverse effects (Diarrhea)**


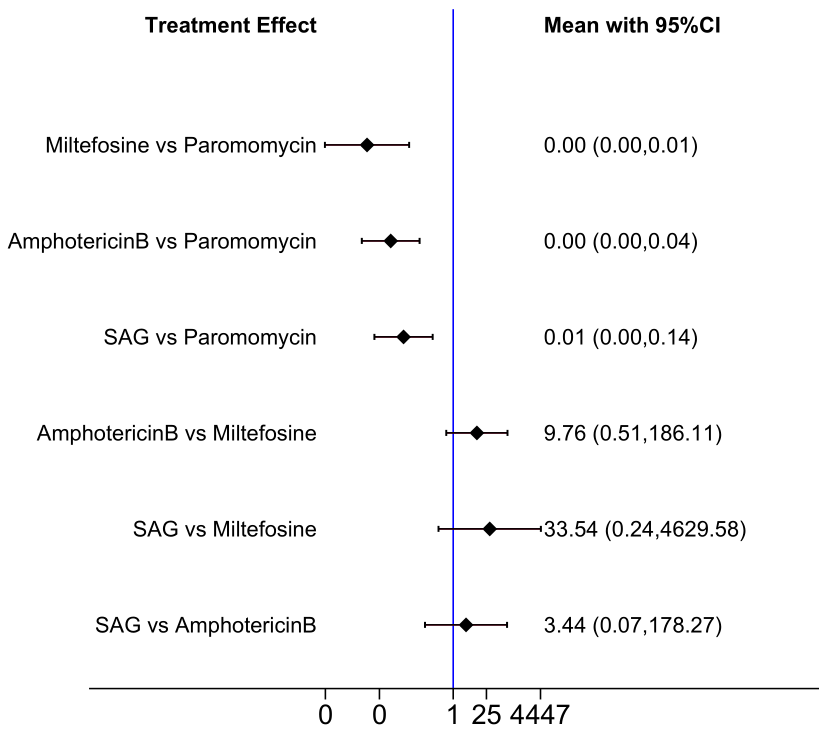


**eFigure 7 Forest plot of network meta-analysis of adverse effects (Injection site pain)**


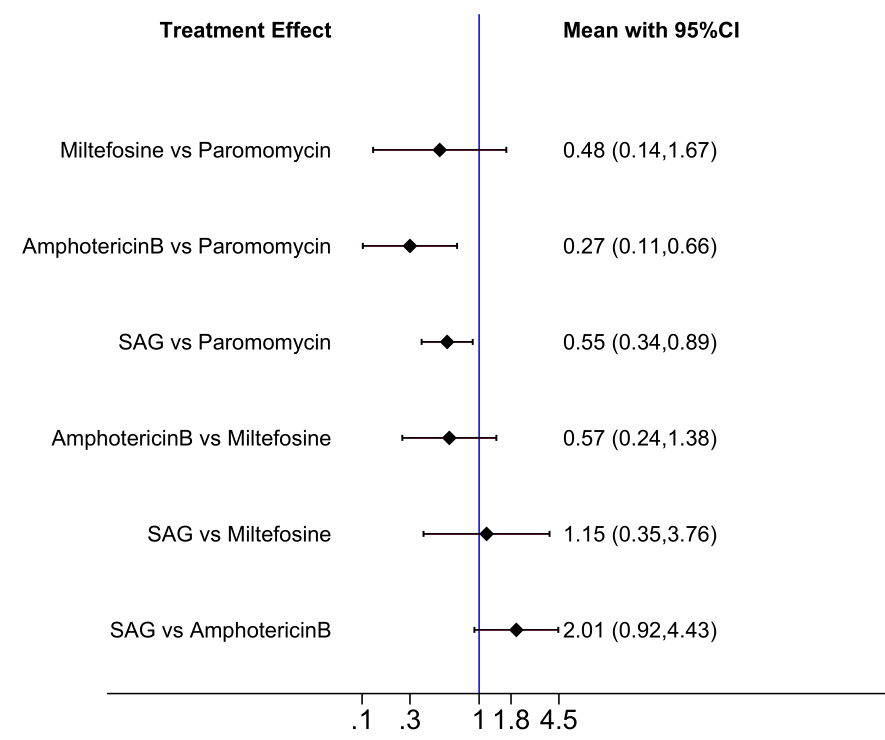


**eFigure 8 Forest plot of network meta-analysis of adverse effects (Abnormal alanine aminotransferase)**


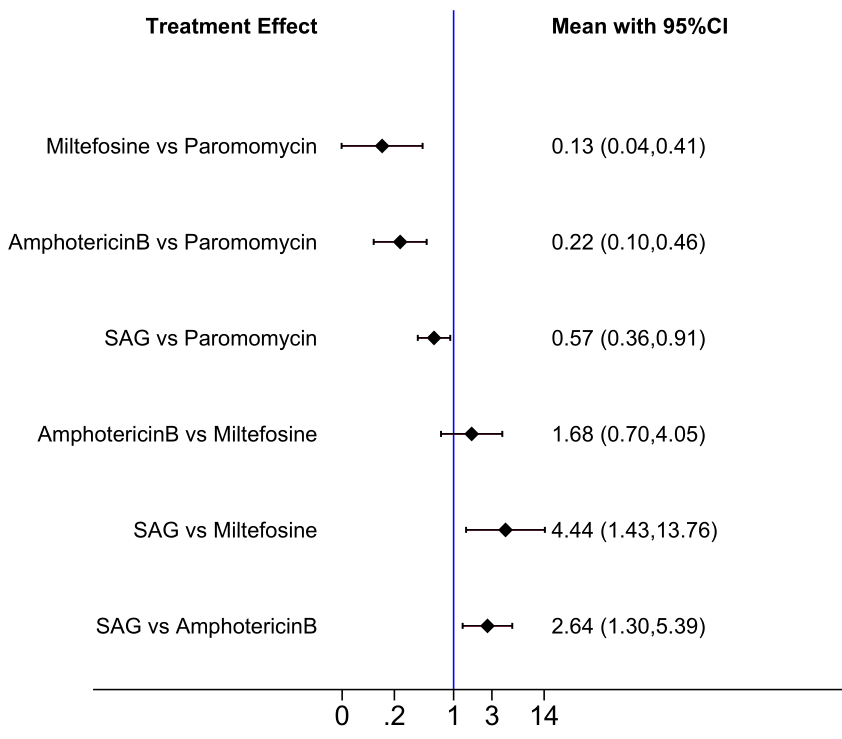


**eFigure 9 Forest plot of network meta-analysis of adverse effects (Abnormal aspartate aminotransferase)**


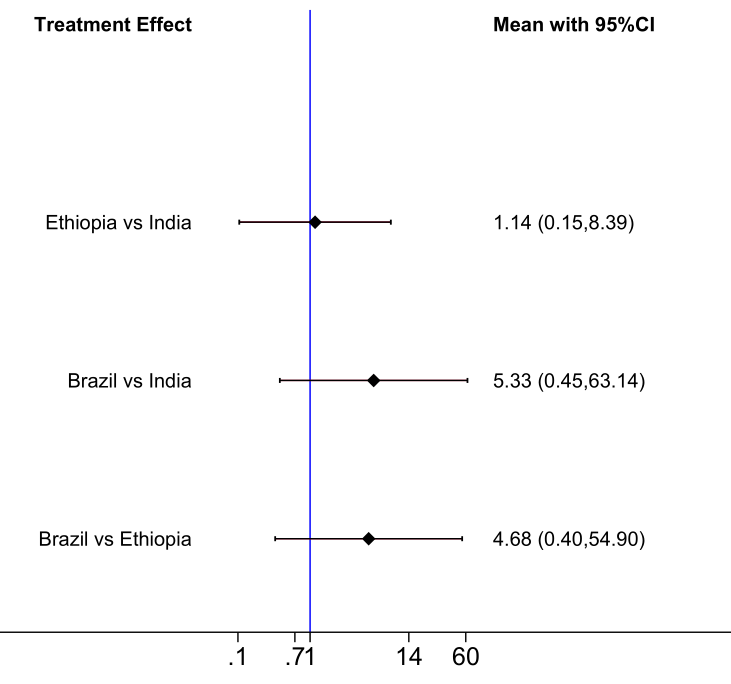


**eFigure 10 Forest map of the efficacy of pentavalent antimony in treating leishmaniasis in different countries**


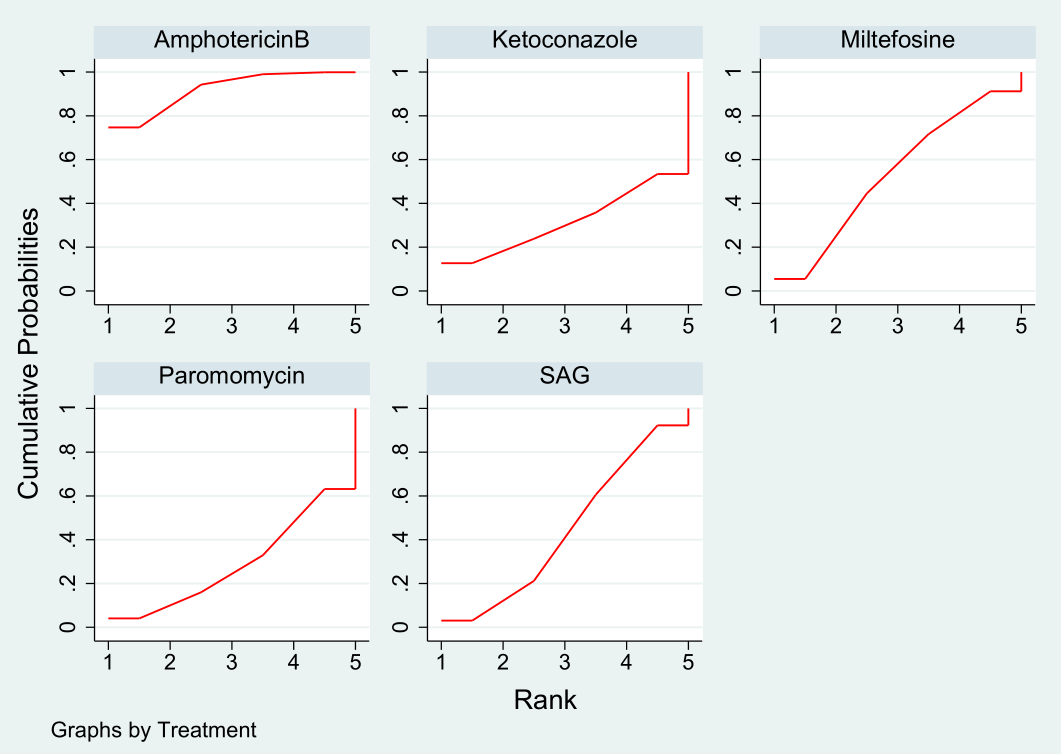


**eFigure 11 SUCRA efficacy ranking curve is clinical cure rate, SUCRA = cumulative ranking surface**


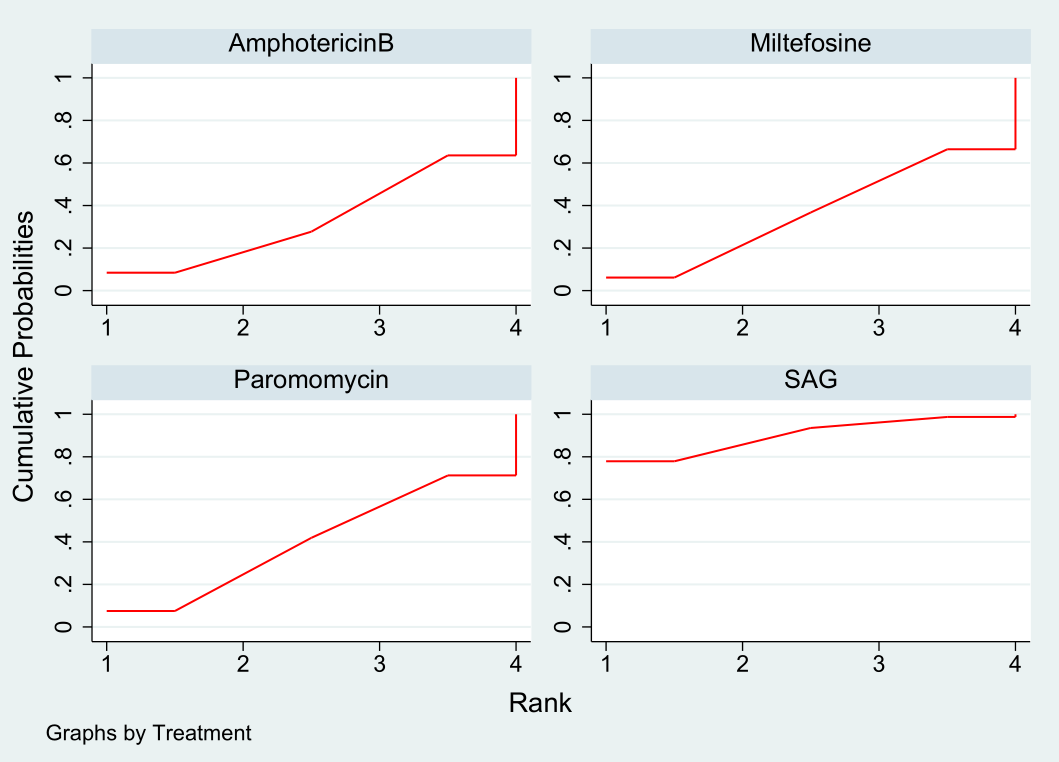


**eFigure 12 SUCRA efficacy ranking curve is mortality, SUCRA = cumulative ranking surface**


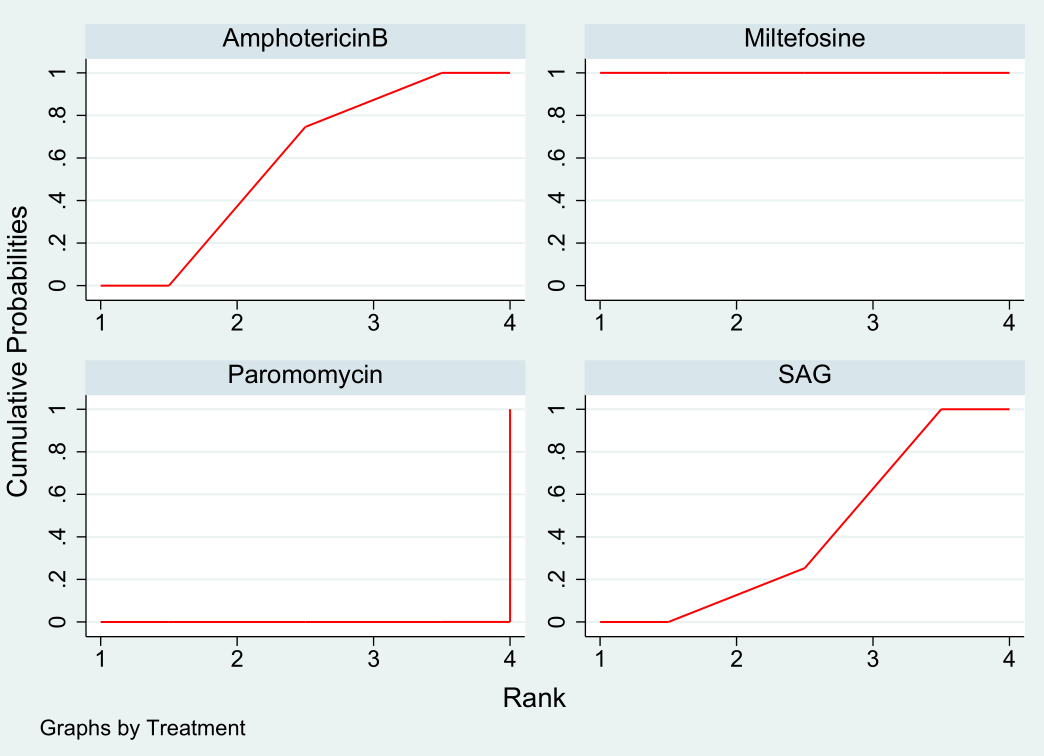


**eFigure 13 SUCRA efficacy ranking curve is the incidence of adverse reactions (Vomiting), SUCRA = cumulative ranking surface**


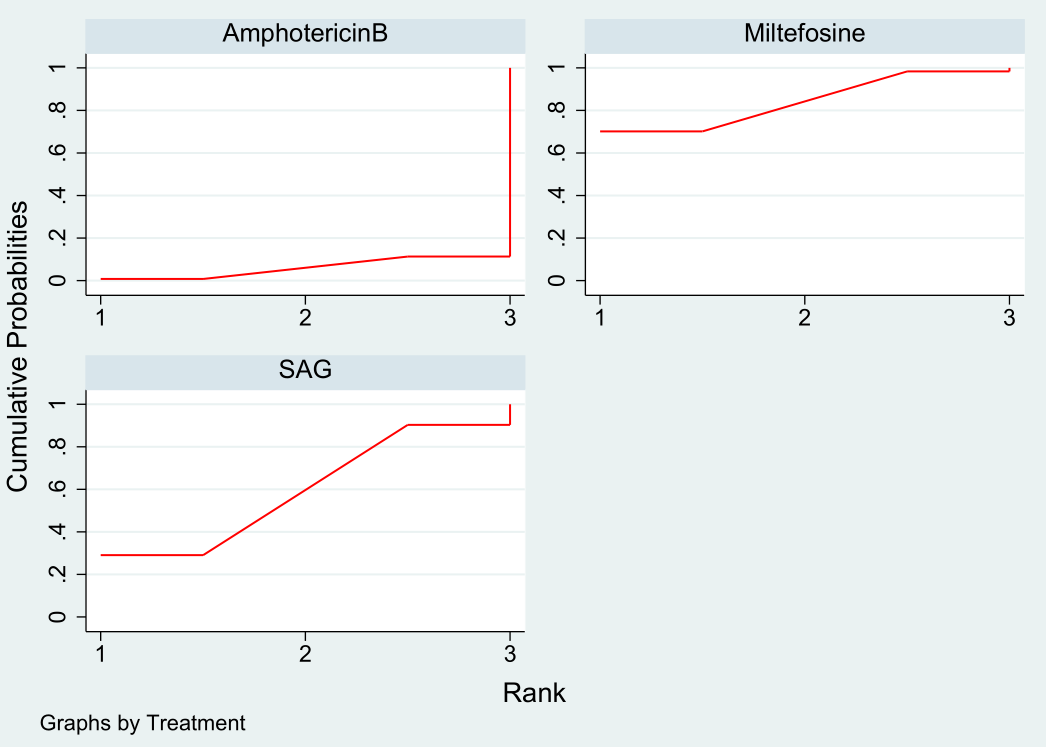


**eFigure 14 SUCRA efficacy ranking curve is the incidence of adverse reactions (Diarrhea), SUCRA = cumulative ranking surface**


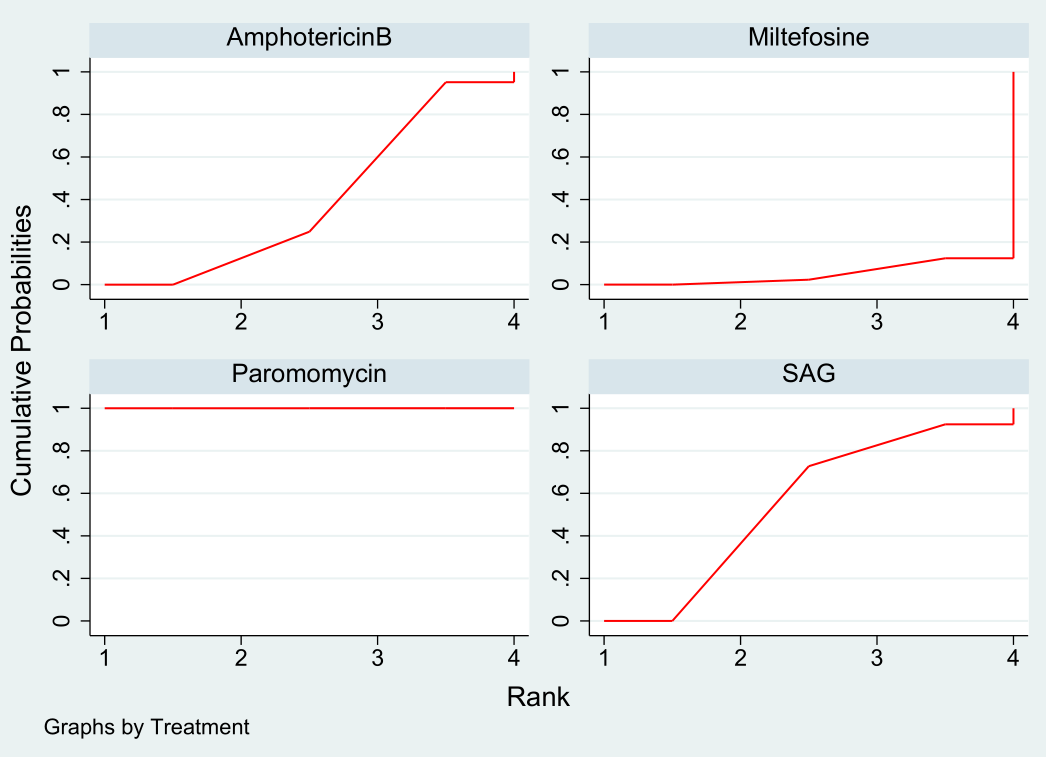


**eFigure 15 SUCRA efficacy ranking curve is the incidence of adverse reactions (Injection site pain), SUCRA = cumulative ranking surface**


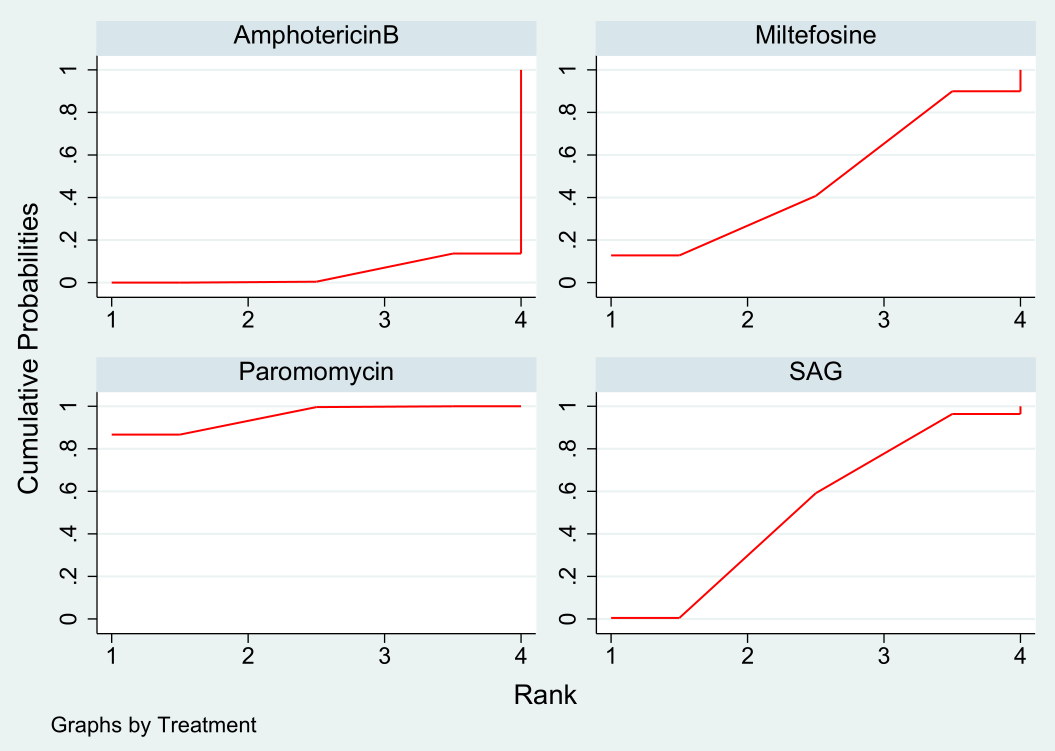


**eFigure 16 SUCRA efficacy ranking curve is the incidence of adverse reactions (Abnormal alanine aminotransferase), SUCRA = cumulative ranking surface**


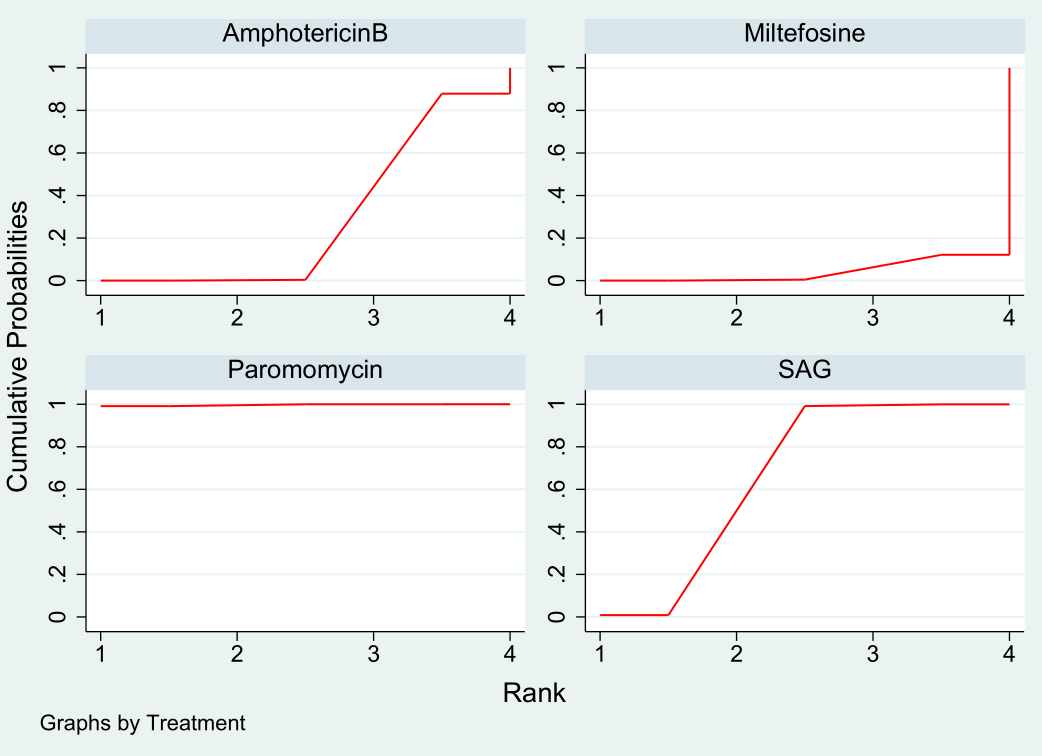


**eFigure 17 SUCRA efficacy ranking curve is the incidence of adverse reactions (Abnormal aspartate aminotransferase), SUCRA = cumulative ranking surface**


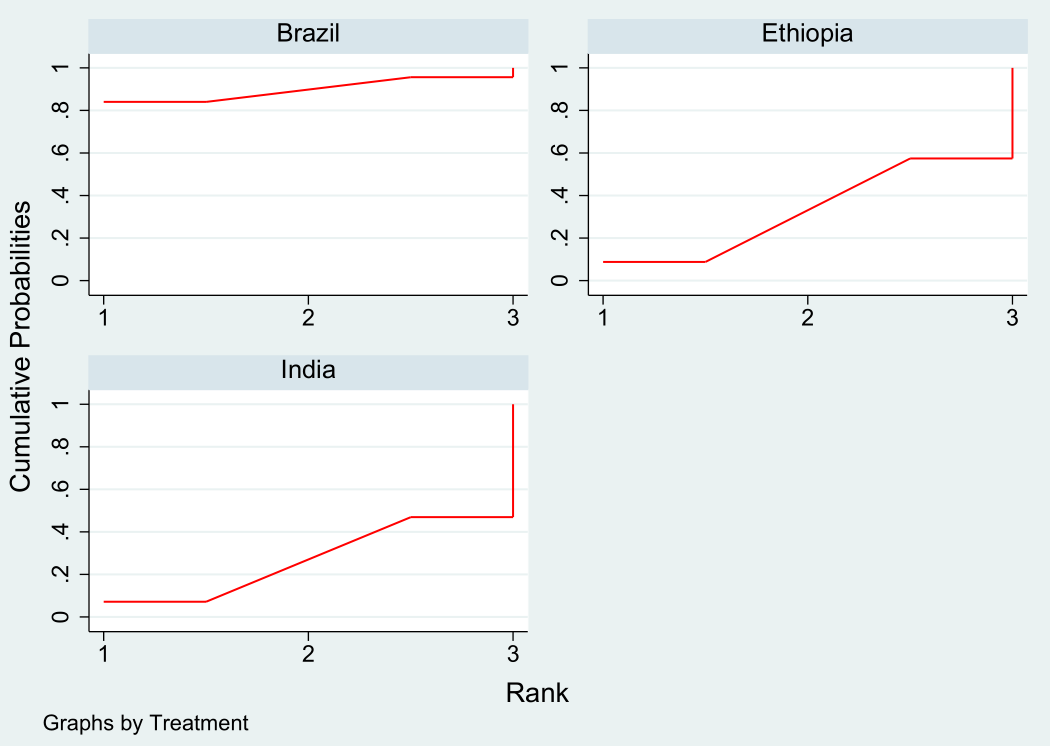


**eFigure 18 SUCRA efficacy ranking curve is the clinical cure rate of leishmaniasis treated with pentavalent antimony in different countries, SUCRA = cumulative ranking surface**


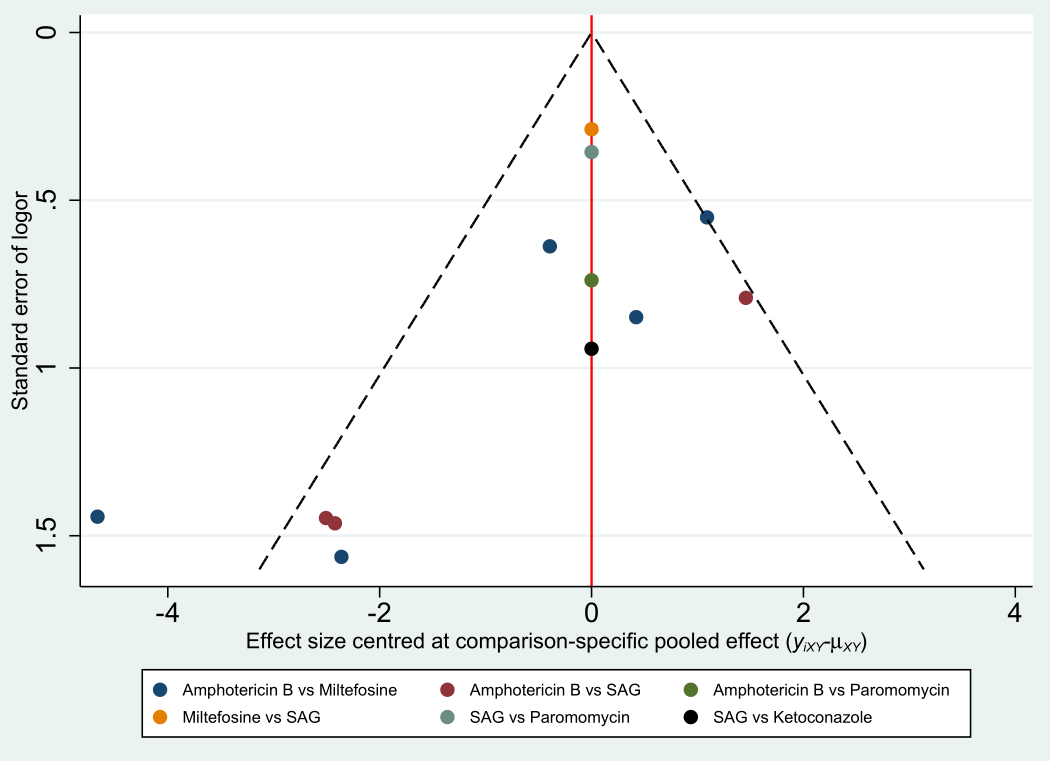


**eFigure 19 Funnel plot of a network meta-analysis of clinical cure rates**


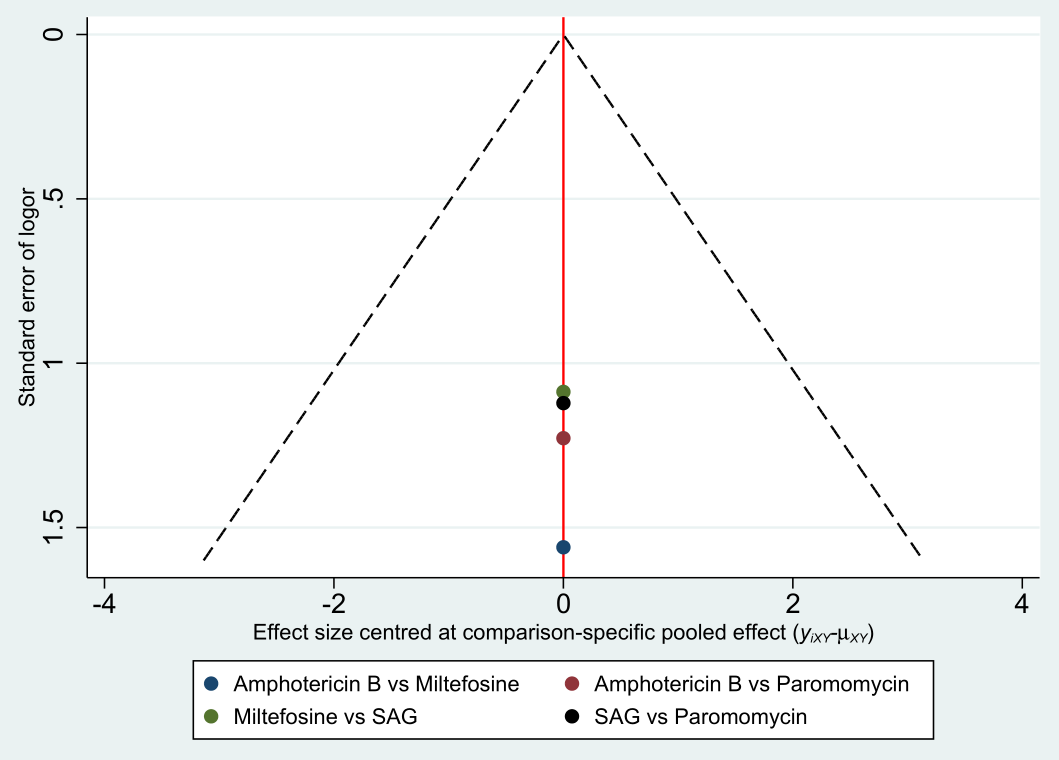


**eFigure 20 Funnel plot of a network meta-analysis of mortality**


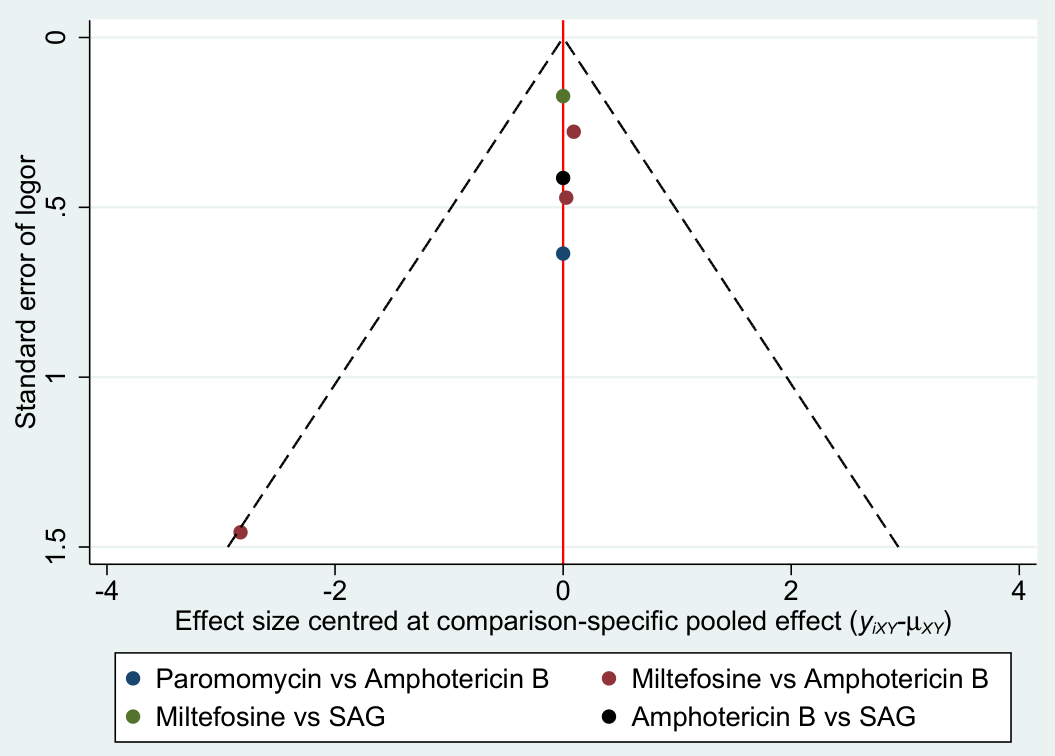


**eFigure 21 Funnel plot of a network meta-analysis of the incidence of adverse events（Vomiting）**


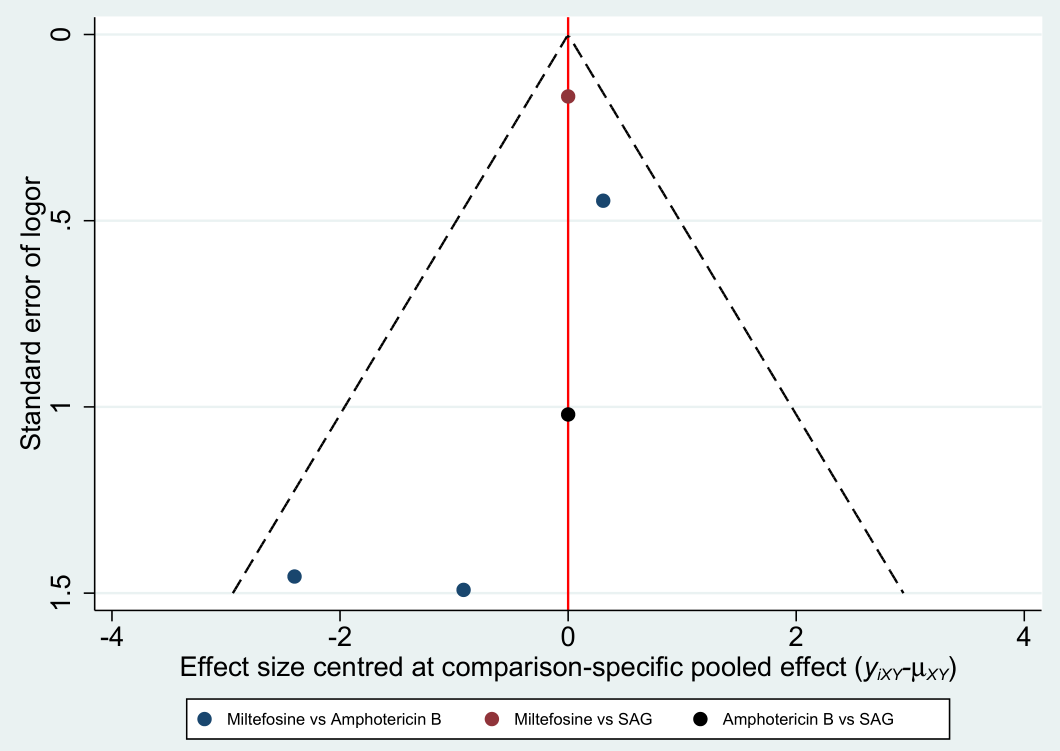


**eFigure 22 Funnel plot of a network meta-analysis of the incidence of adverse events（Diarrhea）**


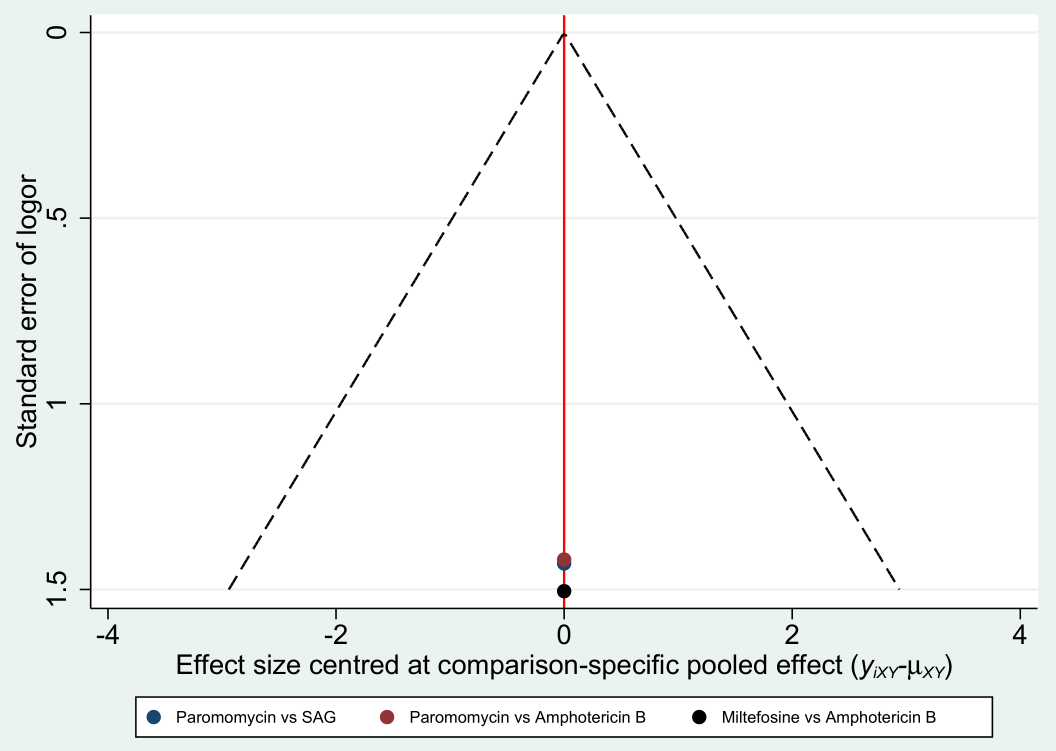


**eFigure 23 Funnel plot of a network meta-analysis of the incidence of adverse events（Injection site pain）**


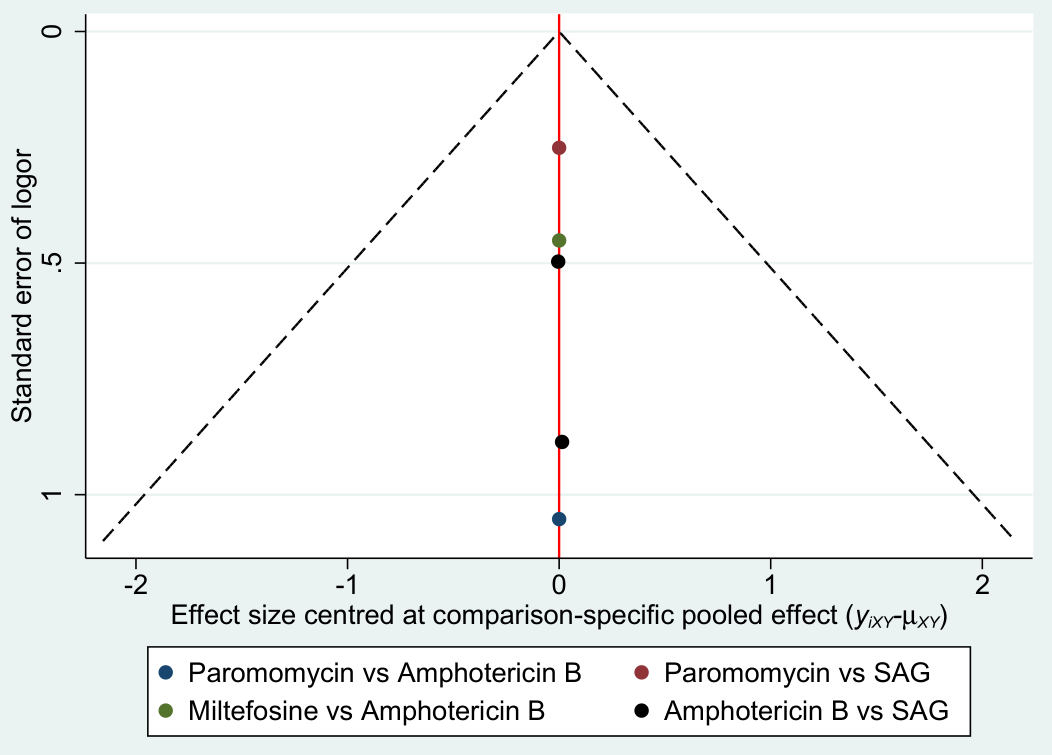


**eFigure 24 Funnel plot of a network meta-analysis of the incidence of adverse events（Abnormal alanine aminotransferase）**


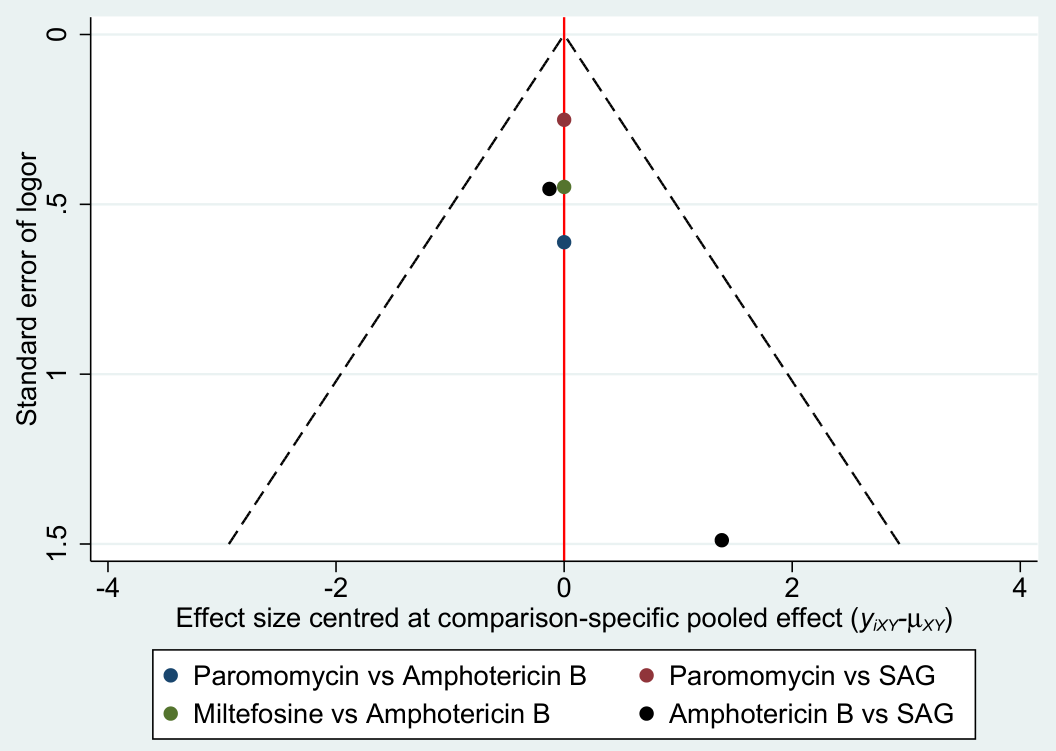


**eFigure 25 Funnel plot of a network meta-analysis of the incidence of adverse events（Abnormal aspartate aminotransferase）**


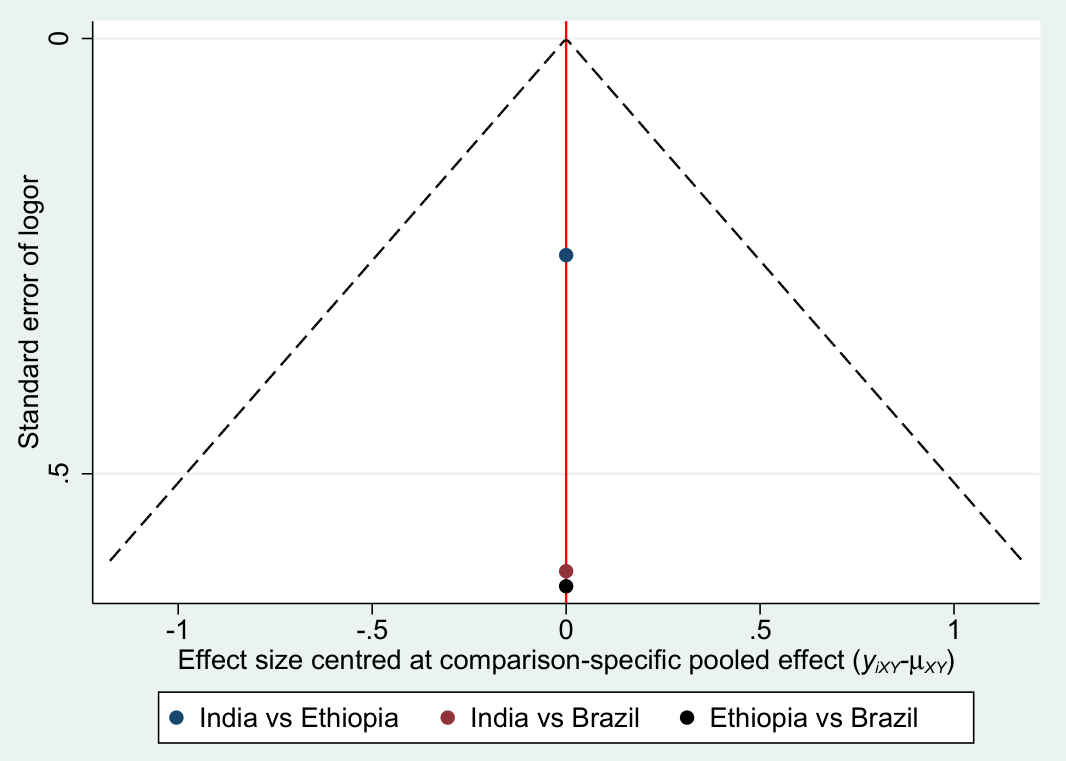


**eFigure 26 Funnel plot of a network meta-analysis of the clinical cure rate of leishmaniasis treated with pentavalent antimony in different countries**
